# Survival of Eurasian lynx in the human‐dominated landscape of Europe

**DOI:** 10.1111/cobi.14439

**Published:** 2025-01-14

**Authors:** J. Premier, M. L. Bastianelli, J. Oeser, O. Anders, H. Andren, M. Aronsson, G. Bagrade, E. Belotti, C. Breitenmoser‐Würsten, L. Bufka, R. Černe, J. Červený, N. Drouet‐Hoguet, M. Ďuľa, C. Fuxjäger, M. Herdtfelder, L. Hočevar, W. Jędrzejewski, R. Kont, P. Koubek, R. Kowalczyk, M. Krofel, J. Krojerová‐Prokešová, J. Kubala, J. Kusak, M. Kutal, J. D. C. Linnell, J. Mattisson, T. L. Middelhoff, D. Melovski, A. Molinari‐Jobin, J. Odden, H. Okarma, A. Ornicāns, N. Pagon, J. Persson, K. Schmidt, M. Sindičić, V. Slijepčević, B. Tám, F. Zimmermann, S. Kramer‐Schadt, M. Heurich

**Affiliations:** ^1^ Chair of Wildlife Ecology and Management Albert Ludwigs University of Freiburg Freiburg Germany; ^2^ Department of National Park Monitoring and Animal Management Bavarian Forest National Park Grafenau Germany; ^3^ Leibniz Institute for Zoo and Wildlife Research (IZW) Berlin Germany; ^4^ Geography Department Humboldt‐Universität zu Berlin Berlin Germany; ^5^ Luchsprojekt Harz Nationalparkverwaltung Harz, Außenstelle Oderhaus Sankt Andreasberg Germany; ^6^ Grimsö Wildlife Research Station, Department of Ecology Swedish University of Agricultural Sciences Riddarhyttan Sweden; ^7^ Latvian State Forest Research Institute Silava Salaspils Latvia; ^8^ Faculty of Forestry and Wood Sciences Czech University of Life Sciences Prague Czech Republic; ^9^ Department of Research and Nature Protection Šumava National Park Administration Kašperské Hory Czech Republic; ^10^ Stiftung KORA Muri bei Bern Switzerland; ^11^ Slovenia Forest Service Ljubljana Slovenia; ^12^ Direction de la Recherche et de l'Appui Scientifique Office Français de la Biodiversité Gières France; ^13^ Department of Forest Ecology, Faculty of Forestry and Wood Technology Mendel University in Brno Brno Czech Republic; ^14^ Luchsbeauftragter Nationalpark Kalkalpen, Nationalpark Zentrum Molln Molln Austria; ^15^ Forstliche Versuchs‐ und Forschungsanstalt Baden‐Württemberg (FVA) Freiburg Germany; ^16^ Biotechnical Faculty University of Ljubljana Ljubljana Slovenia; ^17^ Centro de Ecología Instituto Venezolano de Investigaciones Científicas (IVIC) Caracas Venezuela; ^18^ Mammal Research Institute Polish Academy of Sciences Białowieża Poland; ^19^ Department of Zoology, Institute of Ecology and Earth Sciences University of Tartu Tartu Estonia; ^20^ Institute of Vertebrate Biology Czech Academy of Sciences Brno Czech Republic; ^21^ Department of Zoology, Fisheries, Hydrobiology and Apiculture, Faculty of AgriSciences Mendel University in Brno Brno Czech Republic; ^22^ Department of Applied Zoology and Wildlife Management, Faculty of Forestry Technical University in Zvolen Zvolen Slovakia; ^23^ DIANA – Carpathian Wildlife Research Banská Bystrica Slovakia; ^24^ Department of Biology, Faculty of Veterinary Medicine University of Zagreb Zagreb Croatia; ^25^ Carnivore Conservation Programme Friends of the Earth Czech Republic Olomouc Czech Republic; ^26^ Norwegian Institute for Nature Research Lillehammer Norway; ^27^ Department of Forestry and Wildlife Management, Campus Evenstad Inland Norway University for Applied Science Koppang Norway; ^28^ Norwegian Institute for Nature Research Trondheim Norway; ^29^ Macedonian Ecological Society Skopje North Macedonia; ^30^ Progetto Lince Italia Tarvisio Italy; ^31^ Norwegian Institute for Nature Research Oslo Norway; ^32^ Institute of Nature Conservation Polish Academy of Sciences Krakow Poland; ^33^ Department of Wildlife Management and Nature Protection Karlovac University of Applied Sciences Karlovac Croatia; ^34^ National Zoological Garden Bojnice Bojnice Slovakia; ^35^ Department of Ecology and Evolution University of Lausanne Lausanne Switzerland; ^36^ Chair of Applied Animal Ecology, Institute of Ecology Technische Universität Berlin Berlin Germany

**Keywords:** cause‐specific mortality, compensatory mortality, Eurasian lynx, large carnivore, *Lynx lynx*, survival, carnívoro mayor, línce euroasiático, mortalidad compensatoria, mortalidad por causas espcíficas, supervivencia, *Lynx lynx*

## Abstract

Survival and cause‐specific mortality rates are vital for evidence‐based population forecasting and conservation, particularly for large carnivores, whose populations are often vulnerable to human‐caused mortalities. It is therefore important to know the relationship between anthropogenic and natural mortality causes to evaluate whether they are additive or compensatory. Further, the relation between survival and environmental covariates could reveal whether specific landscape characteristics influence demographic performance. We used telemetry data on 681 Eurasian lynx (*Lynx lynx*), a model apex predator with large spatial requirements, that were tracked across their European distribution. Through time‐to‐event analyses, we sought to determine the variables associated with differences in their survival. Illegal killing was the main cause of mortality (33.8%), and mortality rates were similar in protected and hunted populations (8.6% and 7.0% per year, respectively). Survival varied greatly across populations (70–95% per year). Across all study sites, higher hunting and anthropogenic mortality rates were partially compensated by lower rates of other mortality causes but not by natural mortality alone. Variation in survival depended on sex (female survival was 1.5 times greater than male survival) and seasonality (highest risk during hunting season and winter), and lower survival rates were correlated with higher human modification of landscapes at both coarse (home range composition) and fine (habitat use within home range) scales. Some variation in survival was driven by unobserved factors, which, given the high rates of human‐caused mortalities, including illegal killing, are of foremost concern. Due to the low natural mortality rates in protected and hunted populations, we conclude that anthropogenic causes of mortality are likely close to additive, such that maintaining or increasing refuge habitat with little human disturbance is critical to lynx conservation.

## Abstract (es)

Supervivencia del lince euroasiático en los paisajes europeos dominados por humanos

**Resumen:**

Las tasas de supervivencia y mortalidad por causas específicas son vitales para la previsión y conservación de poblaciones basadas en evidencias, sobre todo en el caso de los grandes carnívoros, cuyas poblaciones suelen ser vulnerables a la mortalidad causada por el hombre. Por esto es importante conocer la relación entre las causas de mortalidad antropogénicas y naturales para evaluar si son aditivas o compensatorias. Además, la relación entre la supervivencia y las covariables ambientales podría revelar si las características específicas del paisaje influyen en el rendimiento demográfico. Utilizamos datos telemétricos de 681 linces euroasiáticos (*Lynx lynx*), un depredador ápice modelo con grandes necesidades espaciales, que fueron rastreados a lo largo de su distribución europea. Mediante análisis cronológicos, intentamos determinar las variables asociadas a las diferencias en su supervivencia. La matanza ilegal fue la principal causa de mortalidad (33.8%), y las tasas de mortalidad auales fueron similares en las poblaciones protegidas y cazadas (8.6 y 7.0%, respectivamente). La supervivencia anual varió mucho de una población a otra (del 70 al 95%). En todos los lugares de estudio, las mayores tasas de mortalidad antropogénica y por caza se vieron parcialmente compensadas por las menores tasas de otras causas de mortalidad, pero no por la mortalidad natural por sí sola. La variación en la supervivencia dependió del sexo (la supervivencia de las hembras fue 1.5 veces mayor que la de los machos) y de la estacionalidad (el mayor riesgo se registró durante la temporada de caza y el invierno), y las menores tasas de supervivencia se correlacionaron con una mayor modificación humana de los paisajes tanto a escala gruesa (composición del área de distribución) como fina (uso del hábitat dentro del área de distribución). Algunas variaciones en la supervivencia se debieron a factores no observados. Dadas las elevadas tasas de mortalidad causada por el hombre, incluidas las matanzas ilegales, estos factores son motivo de gran preocupación. Debido a las bajas tasas de mortalidad natural en poblaciones protegidas y cazadas, concluimos que las causas antropogénicas de mortalidad son probablemente casi aditivas, de modo que mantener o aumentar el hábitat de refugio con poca perturbación humana es crítico para la conservación del lince.

## INTRODUCTION

Survival is a key demographic rate, determined by underlying spatiotemporal distributions of risks and resources (Gaillard et al., 2010). Natural and anthropogenic mortalities vary by period and habitat, which increase or decrease survival probability (DeCesare et al., 2014). This means the survival of an individual should be correlated with the landscapes that they use (Fahrig, 2007). Connecting wildlife mortality events to landscape characteristics can, therefore, potentially reveal how species respond to different pressures (Bastianelli et al., 2021; Oriol‐Cotterill et al., 2015). Conservation of threatened species hinges on understanding the factors, including anthropogenic, that influence survival and causes of mortality (Goodrich et al., 2008). These data are also necessary for evidence‐based engagement with stakeholders in the context of human–wildlife coexistence (Redpath et al., 2013).

Wildlife survival rates in anthropogenic landscapes often depend on direct human actions. Behavioral plasticity can help offset anthropogenic disturbances, for example, by avoiding novel risk factors (Sih, 2013), but large carnivores must confront risks to maintain extensive home ranges that meet their energetic requirements (Carbone et al., 2005). In most cases, home ranges are fully outside protected areas, which could make avoidance of human‐related risks impossible. Moreover, mortality risk can even be high in protected areas (Rauset et al., 2016). As a result, populations can exhibit source–sink dynamics between areas of differing human influences. For example, occurrence of brown bear (*Ursus arctos*) in more human‐influenced areas relies on immigration from less disturbed wilderness areas (Lamb et al., 2020), and pumas (*Puma concolor*) occupying remote areas are a source in more urbanized areas (Nisi et al., 2023).

Habitat selection is a hierarchical process that occurs at different scales to maximize fitness (Johnson, 1980). It is hypothesized that species will avoid the most limiting factors at coarser spatial scales, such as the selection of habitats for their home range composition (hereafter landscape) (Rettie & Messier, 2000). At finer selection scales, such as use of habitat within the home range (hereafter home range), individuals may control the remaining risks spatially and temporally and focus on other basic needs, such as prey resources (Filla et al., 2017; Suraci et al., 2019). However, whether this hierarchy is also reflected in individual fitness is not guaranteed (Basille et al., 2013). To understand the drivers of survival and mortality, they must be studied across gradients of risks and resources and at different spatial scales.

A key concept in wildlife management is that some proportion of individuals in a natural population are destined to die naturally (Errington, 1956). This gives rise to the classic hypothesis that if increases in a given mortality source can be compensated by decreases in other mortality sources, then survival remains constant (i.e., compensatory mortality) (Burham & Anderson, 1984). However, mortality causes can also be additive, whereby increases of a given mortality cause are directly proportional to decreases in survival, or partially compensatory, whereby reductions in other mortality causes do not fully compensate and survival decreases (e.g., Sandercock et al., 2011). Characterizing the relationship between survival and different mortality causes poses a fundamental question in population ecology (Boyce et al., 1999) and is important to ensure sustainable harvest of game species (Marboutin et al., 2003; Medellín, 1999). Due to their ecology, large carnivores are particularly vulnerable to human‐caused mortalities (Krofel et al., 2015), which are often more frequent than natural causes (Moss et al., 2016). In hunted and protected populations, other anthropogenic mortality causes may have additive or compensatory relations to natural mortality (e.g., Murray et al., 2010). By exploring how survival is related to different mortality factors, one can better understand species’ vulnerability to demographic pressures and provide important information for conservation of protected populations and sustainability in hunted populations (e.g., Wolfe et al., 2015).

Assessment of large carnivore survival has typically been conducted on a local scale. Such studies of single populations (e.g., Suutarinen & Kojola, 2017) provide locally relevant information, but their application to interregional questions may be limited. Studies of survival and mortality in multiple populations, or study areas, covering gradients of environmental conditions and management regimes (e.g., Smith et al., 2010; Wolfe et al., 2015) can improve understanding of where large carnivore populations might struggle to coexist with humans (Gilroy et al., 2015). Combining data from different regions can also help improve understanding of the interplay between survival rates and different mortality causes at a species distribution level. These outputs could inform conservation plans at geographic scales that are meaningful for species whose populations cover vast extents, such as large carnivores (Benson et al., 2023).

Telemetry tracking is a precise way to estimate survival in wild animal populations because individuals’ fates are known (Murray & Patterson, 2006) and all mortality causes have equal detection probabilities (Naef‐Daenzer et al., 2017). Analyses of tracking data represent a special subclass in survival modeling, formally called known‐fate models (Heisey & Fuller, 1985), as opposed to methods where mortality must be partially inferred (e.g., camera trapping) (Saracco et al., 2010). Known‐fate models can provide statistically robust estimates of survival and mortality because they consider the number of animals tracked and the length of time they were tracked (Heisey & Fuller, 1985). Multivariate known‐fate modeling has, therefore, frequently been used to relate observed covariates, such as sex and landscape composition, from telemetry locations to survival (Basille et al., 2013; Brodie et al., 2013). The heterogeneity of survival not captured by spatial variation in observed covariates (e.g., effects of human acceptance, wildlife population density) may also contain important information (Halstead et al., 2012). Survival models that can delineate the effects of both observed and unobserved spatially dependent factors (e.g., Zhou & Hanson, 2015) would be advantageous for assessing wildlife survival across large geographic extents because unexplained variability is considered. Thereby, the common drivers, which can inform decision‐making, and variability of unknowns can be better understood.

**FIGURE 1:**
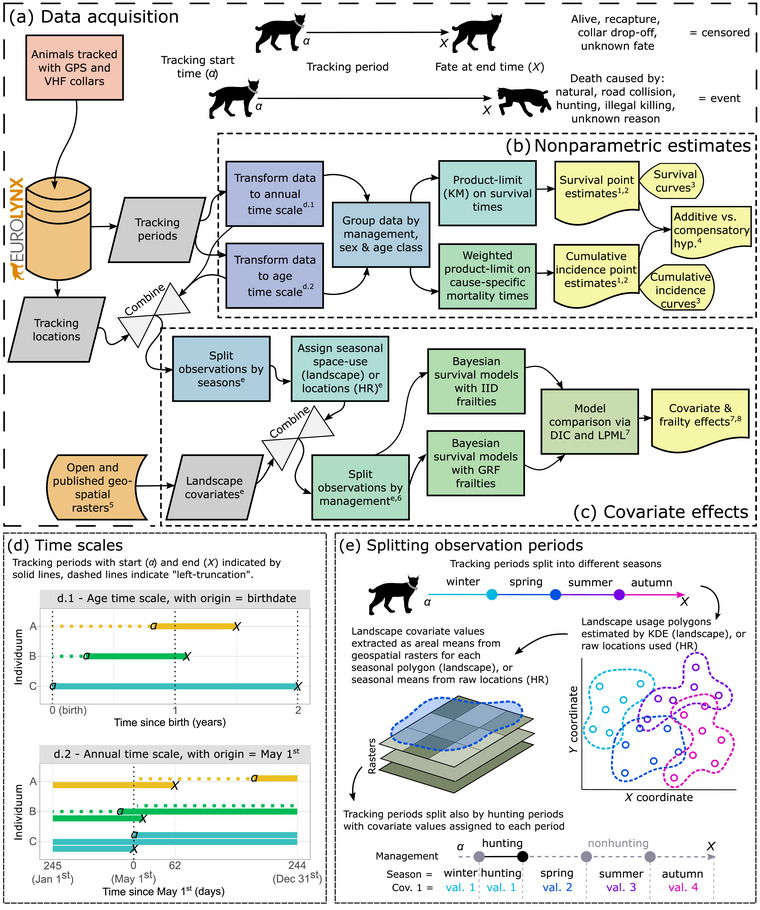
Workflow in a study of Eurasian lynx survival in Europe: (a) acquisition of lynx tracking data and geospatial data (^1^, Table ; ^2^, Appendices S7 & S8; ^3^, Figure 2; ^4^, Figure 3; ^5^, Appendix S5; ^6^, Appendix S1; ^7^, Table 2; ^8^, Table 3 and Appendix S10; ^d^, part [d] of this figure; ^e^, part [e] of this figure; GPS, global positioning system; VHF, very high frequency), (b) tracking period observations (i.e., start time, end time, fate) to provide point estimates and curves of survival and cumulative incidence of cause‐specific mortalities based on product‐limit estimation (i.e., KM, Kaplan–Meier) used also in the analysis of mortality hypotheses, (c) combining tracking periods with location data to model the effects of landscape covariates on survival rates (HR, home range; IID, independent and identically distributed; DIC, deviance information criterion; LPML, log pseudo‐marginal likelihood; GRF, Gaussian random field), (d) transformation of tracking periods to different time scales (d.1, age time scale; d.2, annual time scale), and (e) procedure used to connect season (age time scale), hunting periods (annual time scale), and landscape covariates with observation periods (KDE, kernel density estimation; cov., covariate; val., value).

We compiled telemetry data on 681 Eurasian lynx (*Lynx lynx*) (hereafter lynx), a model apex predator with large spatial requirements, that were tracked across their European distribution (Figure 1a). We used time‐to‐event analyses to provide survival and cumulative incidence of cause‐specific mortality rates for lynx across Europe (Figure 1b). These quantities are vital for evidence‐based population viability analyses. Further, we used these to test for consistency with competing mortality hypotheses, hypothesizing, in line with the compensatory hypothesis, that higher anthropogenic (H1) and hunting mortality (H2) are not associated with lower overall survival and therefore natural mortality declines as anthropogenic (H3) and hunting mortality (H4) declines, and higher hunting mortality is associated with lower alternative mortality causes (H5) and less illegal killing (H6). Further, we aimed to improve species‐level understanding of the factors driving survival with multivariate models (Figure 1c) to inform management as to how to spatially allocate conservation resources more effectively. According to the limiting factor hypothesis in hierarchical habitat selection (Rettie & Messier, 2000), we hypothesized that selected landscape components affect survival in a scale‐dependent manner (H7). At the coarser landscape scale (i.e., home range composition), we hypothesized that human habitat modification and disturbances have a strong negative effect on lynx survival (H8). At the finer home range scale (i.e., within home range habitat use), we hypothesized that survival is less correlated to human‐related landscape characteristics (H9) because these are already avoided at the coarse scale and survival is more positively affected by landscape characteristics indicative of prey resources (H10). Finally, we hypothesized that high‐quality habitat has a positive effect on survival at both spatial scales (H11).

## METHODS

### Data acquisition

We collected tracking data from 21 telemetry studies, representing 9 out of 10 populations occurring in Europe. A total of 681 lynx individuals (314 females, 367 males) were tracked for a combined 1052 years (Appendix S1; Figure 1a). Animal captures and experimental procedures were approved by the government agencies and ethics committees relevant to each study area in accordance with their respective animal welfare laws (details in Appendix S2).

At first capture, 327 (48%) individuals were adults (>2 years), and 115 (17%) and 239 (35%) were subadults (1–2 years) and juveniles (<1 year), respectively. We assigned age classes with high confidence due to the synchronized nature of birth dates (Mattisson et al., 2022), body size development, and social–spatial behaviors (i.e., mating, dispersal). We updated individuals’ age classes over time based on their age, which we calculated from the observed, or estimated, birth date. We set the estimated birth dates as the start date of the birthing season (1 May) (Mattisson et al., 2020) and calculated the year via the individual's age class at the time of their first observation (e.g., Weingarth et al., 2012) or postmortem dental analysis (e.g., Marti & Ryser‐Degiorgis, 2018). Birth years varied from high confidence (natal den visits, camera trapping of females with kittens, postmortem) (54%) to medium confidence (juvenile or subadult at first observation) (23%) to low confidence (adults at first observation, i.e., minimum age) (23%). We conducted all analyses with respect to age class to ensure robust results, and some aspects were repeated with age (see time scales below). We acquired lynx location data, by triangulation of radio signals for very high frequency (VHF) collars, and remote or direct download for global positioning system (GPS) collars, with an average of 1.6 and 3.8 locations/day, respectively. Overall, our data set included 219 mortality events, and the oldest lynxes, based on telemetry tracking observations, were 18.0 and 14.7 years for females and males, respectively.

We investigated potential mortality following triggering of on‐collar mortality sensors or immobility. We categorized telemetry tracking that ended due to mortality into the following causes: natural (disease, inter‐ or intraspecific killing, starvation, falls, and unknown natural causes), illegal killing, legal killing, vehicle collisions (road and train), and unknown (e.g., carcass decomposed or consumed). We included suspected illegal killing following Andrén et al.’s (2006) criteria to distinguish illegal killing from radio transmitter failure and unknown fates (Appendix S3). We also considered the broad groups of anthropogenic (illegal killing, legal killing, and vehicle collisions) and nonhunting (natural, illegal killing, vehicle collisions, and unknown) mortalities for hypotheses H1, H3, and H5. We right‐censored telemetry tracking that ended without mortality (i.e., survival time was not observed because the tracking ended before the animal died; collar was removed, dropped off, or failed).

We transformed the dates and times of the data to comply with 2 different survival time scales (Figure 1d). First, we created a continuous age time scale, which is the natural scale for ageing with the time origin at birth (Lamarca et al., 1998), whereby observations are intervals of age conditional on survival from birth until the first capture, known as left truncation or delayed entry (Geskus, 2011). Second, we created a recurrent annual time scale with origin 1 May (Fieberg & DelGiudice, 2009). The annual time scale splits tracking periods on 1 May so that they represent interval times with a possible range of 0–365 days, regardless of the year. This is useful for estimating annual rates for management types and age classes. We conducted statistical analyses with R 4.1.3 (R Core Team, 2022) and visualized results with the package ggplot2 (Wickham, 2016).

### Nonparametric survival and cause‐specific mortality estimates

Nonparametric methods are appropriate to estimate empirical point estimates and curves of survival rates and cumulative incidence rates of cause‐specific mortality with few assumptions (Figure 1b). We estimated these quantities for sex, management type (legally hunted or protected), and study area. We estimated the survival rates for each year of age based on the age time scale and estimated annual means for subadult and adult age classes based on the annual time scale. We used the product‐limit (i.e., Kaplan–Meier) estimator (Kaplan & Meier, 1958) from the package survival (Therneau, 2021) to estimate survival rates. We used weighted product‐limit estimation to estimate cumulative incidence rates of competing mortalities while accounting for left truncation (Geskus, 2011). For this, with the package mstate (de Wreede et al., 2011), we replicated the data set for each mortality cause and augmented it with time‐dependent truncation weights and censoring weights that accounted for the competing events. We calculated conditional survival rates with the package condsurv (Zabor & Ganon, 2019) and visualized curves with survminer (Kassambara et al., 2017).

### Additive versus compensatory mortality

To assess our predictions regarding the mortality hypotheses (H1–H6), we used nonparametric estimates of annual survival and mortalities for adults only from the study areas where survival rates could be estimated (i.e., survival <1). We fitted a regression model (response vs. predictor) for each hypothesis: H1, survival versus anthropogenic factors (hunting, illegal killing, vehicle collisions); H2, survival versus hunting; H3, natural mortality versus anthropogenic mortality; H4, natural mortality versus hunting mortality; H5, nonhunting mortality versus hunting mortality; and H6, illegal killing versus hunting mortality. For H1 and H2, we predicted regressions with no trend (fully compensatory) (Appendix S4), whereas for H3–H6, we predicted negative trends, which would show a decline in the response mortality rate as their respective predictor mortality increases (compensation). We used beta regression models from betareg (Cribari‐Neto & Zeileis, 2010) because they are suitable for modeling responses in the range 0–1. To account for the uncertainty in the nonparametric estimates, we sampled 10,000 values for each rate from beta distributions (mean and variance defined by each rate's estimate and standard error squared, respectively) and fitted a regression for each sample. Thereafter, we estimated the mean β and 90% highest posterior density intervals (HPD) across samples (Benson et al., 2023) to evaluate H1–H6. For H1 and H2, we assessed the trends and intercepts of the 10,000 regression lines for consistency with the hypotheses: compensatory and overcompensatory (positive trend), additive and superadditive (negative trend with slope <−*S*_0_), and partially compensatory (negative trend but with slope >−*S*_0_), for which we assumed the baseline survival rate (no hunting), *S*_0_, was the intercept of each regression (Appendix S4).

### Multivariate survival modeling

Multivariate models are an appropriate way to make statistical inference about individuals’ mortality risk depending on their exposure to multiple time‐varying and static factors and treatments (e.g., Bradburn et al., 2003). These factors are quantified, or measured, when an individual is observed at certain instances during their monitoring. Between these instances, it is normally assumed that these factors do not change in value and that the risk associated with them during this discrete period is constant (i.e., piecewise constant hazards). In this way, we related observations of lynx's survival and mortality with temporal exposure to landscape features and seasons during their tracking (Figure 1e), as well as their sex. Season is expected to affect survival of lynx (e.g., Andrén et al., 2022); therefore, to accommodate the piecewise constant hazards assumption, we split tracking periods by season (spring, March–May; summer, June–August; autumn, September–November; winter, December–February) and by country‐specific hunting periods (Figure 1e; Appendix S5). Spring coincides with the onsets of the birthing period and natal dispersal of subadults, summer with weaning of kittens, autumn with higher female mobility with kittens, and winter with the onset of mating (Breitenmoser‐Würsten et al., 2007; Mattisson et al., 2022; Samelius et al., 2012; Zimmermann et al., 2005). We fitted candidate models at each spatial scale and survival time scale, divided into 3 sets: base, habitat suitability (HSI), and component models, with different covariates described below.

### Landscape usage and covariates

We used individuals’ locations collected during each season's tracking to characterize their landscape use (i.e., the areas or locations individuals were exposed to over time). These are corollaries to the scales in hierarchical habitat selection (Johnson, 1980), specifically: landscape scale and home range scale (e.g., Oeser, Heurich, Kramer‐Schadt, Mattisson, et al., 2023. For the home range scale, we assigned each individual's locations to seasonal tracking periods by their acquisition times. For the landscape scale, we took all the locations assigned within each individual's seasonal tracking periods and performed kernel density estimation (KDE), implemented in the package amt (Signer et al., 2019); we used the 95% vertices. Similar to Andrén et al. (2022), for periods when individuals had fewer than 25 locations (54%), we used sex‐specific average daily distance traveled as a radius around locations to delineate landscape scale use (Figure 1e). We used different radii for 3 latitudinal groups: above 65°N (female, 4.4 km; male, 6.9 km), from 55 to 65°N (female, 3.2 km; male, 4.6 km), and below <55°N (female, 2.1 km; male, 3.5 km). If an individual had zero locations in a given period, we brought the previous period forward (10% of cases).

We compiled covariates to characterize landscape composition. For the HSI models, we used the HSI map derived in a Europe‐wide study by Oeser, Heurich, Kramer‐Schadt, Mattisson, et al. (2023). For the component model set, we collected variables that corresponded to habitat or anthropogenic influences: forest integrity (index of human modification of forests), greenness variability (vegetation seasonality), topographic ruggedness, land cover (individually as proportions of forest, shrub, grass, crop, and urban land covers), human modification index, accessibility (travel time to cities), distance to major roads, distance to minor roads, distance to settlements, and human population density (Appendix S5). We extracted covariate values at both spatial scales, taking the mean of each for every individual's seasonal tracking period, thereby landscape covariates varied temporally. To avoid potentially problematic correlations between covariates and reduce the dimensionality (component model set), we used principal component analyses (PCAs) and retained principal components (PCs) that explained at least 5% of the variance (Appendix S6) and fitted a model for each, as well as one model with the first 2 PCs that explained the most variance (landscape, 50.3% and 17.5%, respectively; home range, 44.2% and 12.5%, respectively).

To these, we added categorical covariates (base model) parametrically, including sex (time invariant; 2 levels [female as reference and male]) for all models and additional time‐scale‐specific covariates. For the age time scale models, we included season (time varying; 5 levels, autumn, winter, hunting, spring, summer) due to annually varying risk (Fieberg & DelGiudice, 2009). We chose autumn as the reference level due to demographic stability typical in this season (e.g., Weingarth et al., 2015). We assigned hunting season to individuals exposed to this risk by splitting tracking periods at the start and end of the country‐specific hunting periods (Appendix S1) and replacing the calendar season within this period with the level of hunting (Figure 1e). For the annual time scale models, we included age class (time varying; 3 levels, juvenile [reference], subadult, adult) and hunting period (time varying; 2 levels, hunting [reference], nonhunting), whereby we assigned the level nonhunting to all tracking periods outside hunting periods.

### Model fitting and comparison

To determine the effects of time‐varying covariates at different spatial scales on survival (H7–H11), we fitted multivariate Bayesian semiparametric accelerated failure time models (Zhou & Hanson, 2018) from the package spbayessurv (Zhou et al., 2020). This is a powerful method that accommodates arbitrary censoring, left truncation, time‐varying covariates, and geographic information without the constraint of proportional hazards. In the absence of precise information, we used noninformative default priors. We fitted models with adaptive Markov chain Monte Carlo sampling methods, comprising an initial parametric phase with a lognormal centering distribution (5000 draws) that provides a guide to the baseline survival function in the main semiparametric phase (5000 burn‐ins, retaining 10,000 draws from 50,000). We included frailty terms, which are equivalent to random effects, to account for unobserved factors that influenced survival and to address the lack of independence of individuals with repeated observations (annual time scale) and multiple individuals in the same study area (age and annual time scales). Specifically, we used independent and identically distributed (IID) frailties for nonspatially referenced models, where observations are grouped by individual and study area, and Gaussian random field (GRF) frailties for spatially explicit models, where variation of risk depends on a multivariate spatial distribution. We used the centroids of individuals’ locations as the input coordinates for the GRF frailty.

We fitted each model set once for each frailty (GRF, IID), survival time scale (age, annual), and spatial scale (landscape, home range) for a total of 64 models. We used the deviance information criterion (DIC) and the log pseudo‐marginal likelihood (LPML), where smaller DIC and larger LPML indicate better model performance, to select the most parsimonious models (ΔDIC or ΔLPML <2) in each time and spatial scale combination (16 candidate models each). We checked model performance of selected models visually via conditional Cox–Snell residuals (Zhou & Hanson, 2018). To determine coefficient effects, we estimated their evidence ratios relative to zero for the continuous covariates and among different levels of the categorical covariates with the package brms (Bürkner, 2017).

## RESULTS

### Survival estimates and cause‐specific mortality estimates

**FIGURE 2:**
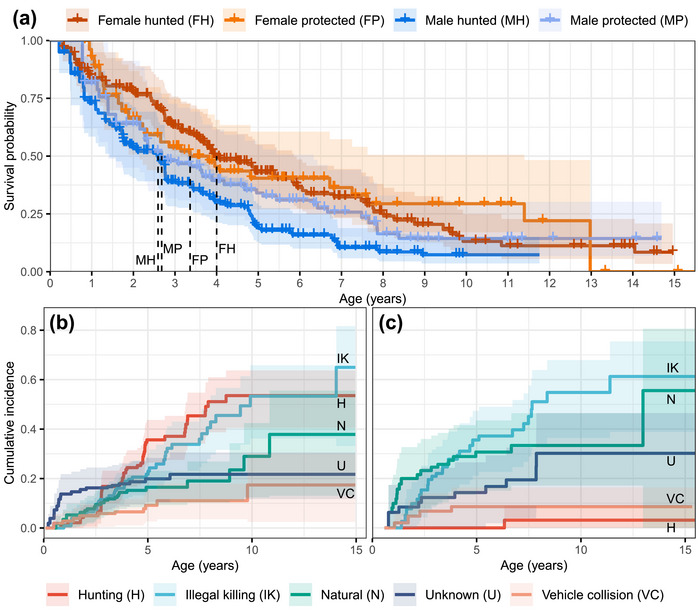
For Eurasian lynx in Europe, (a) product‐limit (Kaplan–Meier) survival estimates for sex and management groups (dotted lines, median survival ages) and weighted product‐limit (Geskus, 2011) cumulative incidence estimates of cause‐specific mortalities of (b) lynx subject to hunting and (c) protected lynx (no legal hunting besides extenuating circumstances). Tabularized values are in Appendices S7 and S8.

Overall, the median survival age (i.e., age when survival probability reaches 0.5) of males was 2.59 (95% confidence interval [CI] 1.43–3.43) and 2.68 (95% CI 1.39–6.33) years in hunted and protected populations, respectively, and for females, it was 4.00 (95% CI 3.28–5.85) and 3.36 (95% CI 2.15–11.39) years, respectively (Figure 2a). The lifespans of females and males in protected populations were generally longer than their hunted counterparts, though annual adult survival had a large range from 0.70 (Harz Mountains) to 0.95 (Dinaric southeastern Alps). In summary, the point estimates showed that hunting and illegal killing were the most important mortality causes for lynx and the incidence of illegal killing in protected and hunted populations was almost equal to the incidence of hunting mortality in hunted populations (point estimates, Table 1; curves, Figure 2; detailed groups, Appendices S7 & S8).

**TABLE 1:** Annual nonparametric estimates of Eurasian lynx survival and cumulative incidence of competing cause‐specific mortality events for a subset of groups.

Parameter	Age class	Sex	Management or other	Estimate (95% CI)
Survival	Adult	Female	Hunted	0.82 (0.78–0.86)
			Protected	0.86 (0.79–0.91)
		Male	Hunted	0.76 (0.70–0.80)
			Protected	0.83 (0.76–0.88)
	Subadult	Female	Hunted	0.93 (0.83–0.97)
			Protected	0.72 (0.50–0.85)
		Male	Hunted	0.73 (0.58–0.84)
			Protected	0.78 (0.55–0.90)
Natural	Adult	Female	Protected	0.031 (0.0010–0.060)
		Male	Protected	0.043 (0.0090–0.077)
		Mean	Hunted	0.033 (0.017–0.048)
			Protected	0.038 (0.014–0.061)
	Subadult	Mean	Hunted	0.037 (0.0010–0.071)
			Protected	0.111 (0.014–0.19)
Illegal killing	Adult	Female	Protected	0.081 (0.031–0.13)
		Male	Protected	0.089 (0.041–0.13)
		Mean	All populations	0.074 (0.056–0.091)
			Hunted	0.070 (0.048–0.091)
			Protected	0.086 (0.051–0.12)
			Reintroduced	0.079 (0.041–0.12)
	Subadult	Mean	Hunted	0.050 (0.0060–0.092)
			Protected	0.098 (0.0020–0.186)
Hunting	Adult	Female	Hunted	0.055 (0.028–0.080)
		Male	Hunted	0.15 (0.10–0.20)
Vehicle		Mean	Hunted	0.012 (0.002–0.022)
			Protected	0.012 (0–0.025)
	Subadult	Mean	Hunted	0.022 (0–0.051)
			Protected	0.048 (0–0.11)

*Note*: For nonparametric estimates calculated for groups not shown, see Appendices S7 and S8.

Abbreviation: CI, confidence interval.

### Additive versus compensatory mortality

**FIGURE 3:**
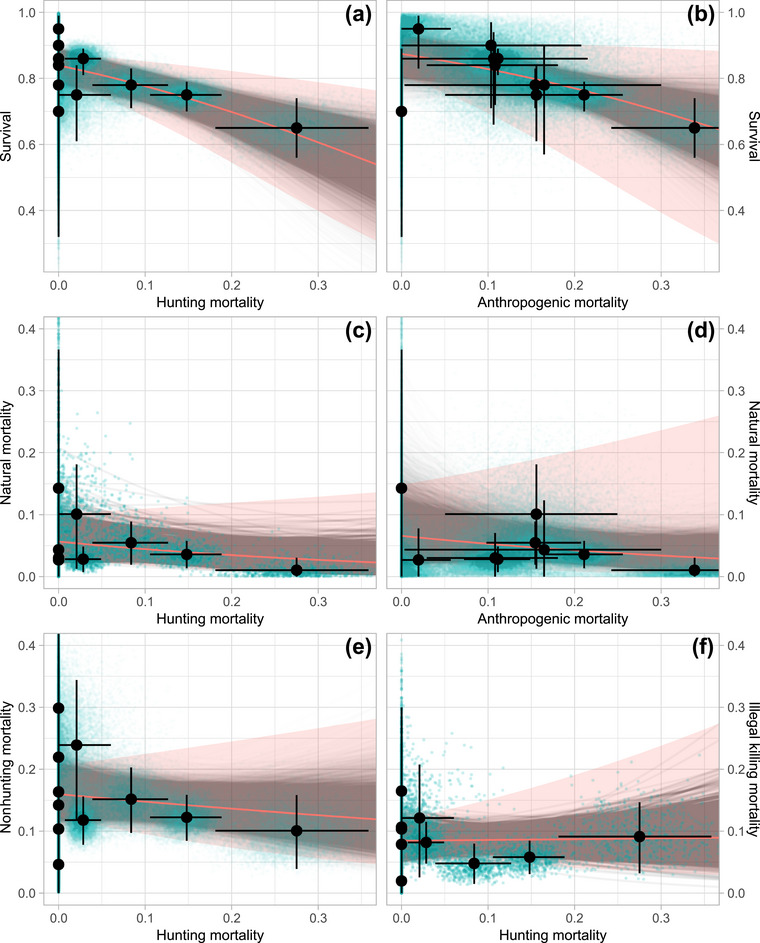
The relationships between Eurasian lynx (a, b) survival and (c, d) natural mortality with hunting mortality and anthropogenic mortality, respectively, and the relationship between (e) nonhunting mortality and (f) illegal killing, with hunting mortality used to test the hypotheses of compensatory mortality (H1–H6) (black dots, nonparametric point estimates; vertical and horizontal lines, 95% confidence interval of adult annual survival and annual cumulative incidence mortality rates, respectively; red lines, mean regressions; pink, 90% highest posterior density intervals [HPD] based on 10,000 samples [blue dots]; gray lines, regression of each sample).

In contrast to our hypotheses (H1 & H2), there was no evidence of compensatory mortality, as annual adult survival decreased with increasing anthropogenic mortality (β = −3.67, 90% HPD −6.23 to −1.22) and hunting mortality (β = −4.08, 90% HPD −5.90 to −2.01) (Figure 3a,b). Instead, these results support the additive or partially compensatory mortality hypotheses because survival was negatively influenced by anthropogenic and hunting mortality. Based on the 10,000 sampled regressions, 22% and 46% of the regression slopes for anthropogenic and hunting mortality against survival, respectively, were steep enough to fit the additive mortality hypothesis. The remaining majority were consistent with partially compensatory mortality (Appendix S9). We found negative relationships between natural mortality and anthropogenic (β = −2.16, 90% HPD −6.11 to 2.22) and hunting (β = −2.44, 90% HPD −5.46 to 0.946) mortality rates (Figure 3c,d). However, their HPD intervals overlapped zero and, therefore, provided no evidence for the compensatory mortality we predicted (H3 & H4). This suggests that anthropogenic and hunting mortalities were not, or only partially, compensated by changes in natural mortality. Indeed, adult natural mortality rates were similar in hunted and protected populations (Table 1). The posterior mean regression of nonhunting mortality with hunting mortality showed a negative trend (β = −0.944, 90% HPD −2.87 to 1.21), consistent with H5. There was a slight positive trend in the regression between mortality due to illegal killing and hunting mortality (β = 1.11, 90% HPD −1.72 to 5.25), in opposition to H6. However, in both cases, the regressions were statistically inconclusive because their HPD intervals overlapped zero (Figure 3e,f). In summary, increases in hunting mortality were not directly proportional to reductions of any single mortality cause or the combination of all nonhunting mortalities across the different study areas. This suggests that populations were only partially able to compensate for hunting mortality.

### Covariate effects

Across all selected models, we found very strong evidence that male survival times were lower than female survival times (30–36% less) (Table 3). Naturally, survival declined as lynx aged; the highest hazard rates were from 1 to 3 years (Appendices S12 & S13). Considering seasonality, there was very strong evidence across models that hunting season reduced survival probabilities more than any calendar season. The age time scale models offered strong evidence that survival was lower during winter compared with autumn and summer but only moderate evidence compared to spring. Based on the age time scale models, lynx survival probability declined 3 times faster during hunting season (typically winter) and 1.7 times faster during winter (without hunting) than during autumn. There was weak to no evidence that spring, summer, and autumn differed. The annual time scale models reflected these results with hazard rates generally increasing in winter (Appendices S14 & S15). Considering the selected models fitted with the annual time scale, there was only a little evidence (ratios 4–6) that juvenile, subadult, and adult lynx differed in annual survival probabilities. This is likely due to the smaller sample size of younger lynx and because the annual time scale does not consider senescence, which can cause bias, especially for the adult age class (Koons et al., 2014), whereas the alternative age time scale showed senescence naturally.

At the landscape scale, several models with landscape covariates were selected (Table 2), but most found only little to no evidence that they affected survival rates (Table 3). There was moderate evidence from one model on the annual time scale that PC1, which primarily described a gradient from human‐dominated areas to areas with low accessibility and great distance from human infrastructure (Appendix S16), was associated with higher survival times. Higher values of PC1, and therefore home range composition with little anthropogenic influence on average, increased survival probability (H8). Although one HSI model was selected, there was little evidence that the variable affected survival at this spatial scale (H11).

**TABLE 2:** Comparisons of multivariate models of survival of Eurasian lynx for selected^a^ and base^b^ candidate models for each spatial scale and survival time scale^c^.

Scale	Model	DIC^d^	ΔDIC	LPML^e^	ΔLPML
Landscape, age	Comp. GRF, PC5	6438.70	0.00	−3229.46	1.96
	Comp. GRF, PC1	6439.16	0.46	−3227.50	0.00
	Comp. GRF, PC4	6439.70	0.99	−3228.75	1.25
	HSI GRF	6439.84	1.13	−3229.36	1.86
	Base GRF	6444.21	5.51	−3228.37	0.87
	Base IID	6450.44	11.74	−3232.33	4.83
Landscape, annual	Comp. GRF, PC5	3455.60	0.00	−1733.87	4.00
	Comp. GRF, PC4	3455.93	0.33	−1729.87	0.00
	Comp. GRF, PC1	3457.24	1.64	−1731.12	1.26
	Base GRF	3457.94	2.33	−1731.14	1.27
	Base IID	3465.11	9.51	−1735.86	5.99
Home range, age	Comp. GRF, PC1	6434.54	0.00	−3227.12	0.24
	Comp. GRF, PC1 + 2	6436.71	2.16	−3227.84	0.96
	Base GRF	6436.98	2.44	−3226.88	0.00
	Comp. GRF, PC4	6441.09	6.55	−3228.04	1.15
	Base IID	6446.83	12.28	−3232.73	5.85
Home range, annual	HSI GRF	3452.99	0.00	−1729.59	0.00
	Comp. GRF, PC2	3453.24	0.25	−1730.79	1.19
	Comp. GRF, PC1	3454.92	1.93	−1730.87	1.27
	Base GRF	3461.04	8.05	−1733.28	3.69
	Base IID	3465.16	12.17	−1736.30	6.71

Abbreviations: Comp., component; GRF, Gaussian random field frailties; HSI, habitat suitability index; IID, independent and identically distributed frailties; PC, principal component.

^a^ Models selected with change in deviance information criterion (ΔDIC) or change in log pseudo‐marginal likelihood (ΔLPML) <2. Comparison of all candidate models is in Appendix S10.

^b^ Base covariates only: sex + season (age time scale) and sex + hunting + age class (annual time scale).

^c^ Landscape and home range spatial scales and age and annual survival time scales.

^d^ Deviance information criterion. The smaller the DIC, the better the model quality. Models ordered by increasing DIC.

^e^ Log pseudo‐marginal likelihood. The larger the LPML, the better the model performance.

**TABLE 3:** Covariate effect estimates from selected^a^ multivariate Eurasian lynx survival models for each spatial and time scale with estimates from the most parsimonious model in which each covariate appeared^b^.

Scale	Model	Term	Estimate^c^	90% CI^d^	Test^e^	Evidence^f^	*p* ^g^
Landscape, age	Comp. GRF, PC5	β sex: male	0.410	0.188 to 0.633	>female^h^	1249	0.999
		β season: hunting	1.12	0.734 to 1.59	>autumn^h^	Inf	1.00
			0.645	0.236 to 1.12	>winter	302	0.996
			1.20	0.613 to 1.98	>spring	4999	0.999
		β season: spring	0.320	−0.187 to 0.837	>autumn^h^	6.11	0.859
			0.178	−0.476 to 0.743	>summer	2.35	0.701
		β season: summer	−0.0548	−0.470 to 0.377	<autumn^h^	1.48	0.598
		β season: winter	0.647	0.243 to 1.11	>autumn^h^	399	0.997
			0.558	0.00911 to 1.19	>spring	19.2	0.950
			0.736	0.263 to 1.21	>summer	139	0.992
		β PC5	−0.00281	−0.209 to 0.203	<intercept	1.05	0.512
		φ GRF frailty scale	0.00423	0.00149 to 0.00925			
		τ^2^ frailty variance	0.378	0.143 to 0.759			
	Comp. GRF, PC1	β PC1	−0.0280	−0.125 to 0.0842	<intercept	2.17	0.684
	Comp. GRF, PC4	β PC4	0.0884	−0.0466 to 0.224	>intercept	6.34	0.863
	HSI GRF	β HSI	0.00491	−0.00380 to 0.0152	>intercept	3.88	0.795
Landscape, annual	Comp. GRF, PC5	β age class: subadult	−0.359	−1.00 to 0.276	<juvenile^h^	4.45	0.816
		β age class: adult	−0.354	−1.00 to 0.240	<juvenile^h^	4.89	0.830
			0.00494	−0.405 to 0.405	>subadult	1.06	0.515
		β sex: male	0.337	0.0856 to 0.589	>female^h^	77.1	0.987
		β hunt. P^i^.: nonhunt.	−1.64	−4.33 to −0.632	<hunting^h^	Inf	1.00
		β PC5	−0.0168	−0.233 to 0.209	<intercept	1.28	0.562
		φ GRF frailty scale	0.00306	0.000808 to 0.00648			
		τ^2^ frailty variance	0.634	0.200 to 1.31			
	Comp. GRF, PC4	β PC4	0.125	−0.00313 to 0.261	>intercept	17.3	0.945
	Comp. GRF, PC1	β PC1	−0.0733	−0.142 to −0.00489	<intercept	25.5	0.962
	Base GRF	–					
Home range, age	Comp. GRF, PC1	β sex: male	0.400	0.158 to 0.659	>female^h^	269	0.996
		β season: hunting	1.10	0.710 to 1.56	>autumn^h^	Inf	1.00
			0.554	0.195 to 0.953	>winter	255	0.996
			0.375	−0.0385 to 0.845	>spring	12.8	0.927
		β season: spring	0.177	−0.283 to 0.624	>autumn^h^	2.82	0.738
			0.287	−0.188 to 0.727	>summer	5.25	0.840
		β season: summer	−0.110	−0.527 to 0.293	<autumn^h^	2.06	0.674
		β season: winter	0.553	0.141 to 1.01	>autumn^h^	80.9	0.987
			0.375	−0.0385 to 0.845	>spring	12.8	0.927
			0.663	0.255 to 1.09	>summer	587	0.998
		β PC1	−0.118	−0.224 to −0.0185	<intercept	49.7	0.980
		φ GRF frailty scale	0.00323	0.000508 to 0.00788			
		τ^2^ frailty variance	0.607	0.222 to 1.50			
	Comp. GRF, PC1 + 2	β PC2	0.00440	−0.0847 to 0.0875	>intercept	1.25	0.556
	Base GRF	–					
	Comp. GRF, PC4	β PC4	−0.0881	−0.182 to 0.0114	<intercept	13.0	0.928
Home range, annual	HSI GRF	β age class: subadult	−0.360	−0.978 to 0.211	<juvenile^h^	5.57	0.847
		β age class: adult	−0.304	−0.828 to 0.213	<juvenile^h^	4.78	0.827
			0.0555	−0.304 to 0.422	>subadult	1.44	0.591
		β sex: male	0.340	0.110 to 0.587	>female^h^	105	0.990
		β hunt. P.: nonhunt.	−1.01	−2.04 to −0.478	>hunting^h^	Inf	1.00
		β HSI	−0.00773	−0.0167 to 0.00108	<intercept	12.7	0.927
		φ GRF frailty scale	0.00181	0.000598 to 0.00392			
		τ^2^ frailty variance	0.846	0.282 to 1.75			
	Comp. GRF, PC2	β PC2	0.0597	−0.0323 to 0.159	>intercept	6.14	0.860
	Comp. GRF, PC1	β PC1	−0.122	−0.208 to −0.0515	<intercept	453	0.997

Abbreviations: Comp., component; GRF, Gaussian random field frailties; HSI, habitat suitability index; IID, independent and identically distributed frailties; PC, principal component.

^a^ Candidate models with change in deviance information criterion (ΔDIC) or change in log pseudo marginal likelihood (ΔLPML) <2 selected (Table 2).

^b^ Covariate estimates for each scale shown for the most parsimonious selected model via ΔDIC. Models ordered by increasing DIC. Full output in Appendix S11.

^c^ Positive and negative β coefficients indicate accelerated (i.e., shorter) and decelerated (i.e., longer) survival times, respectively, where $e^{-\beta }$ gives the multiplicative change in median survival time per unit covariate increase (acceleration factor). Within terms, the scale parameter, φ, describes the rate of decay in spatial correlations via $1-e^{\left(-\varphi |\mathrm{distance}|\right)}$, and τ^2^ is the variance in the survival time in frailty (i.e., random effect).

^d^ Credible intervals of coefficients.

^e^ The test indicates one‐sided hypothesis tests used to estimate evidence ratios and posterior probabilities of the statements (posterior distributions [Appendices S22–S25]).

^f^ The ratio between the probability the test is true and that it is false. Evidence close to 1 indicates a low likelihood that coefficients met the test hypothesis.

^g^ Posterior probability of the estimate.

^h^ Reference category.

^i^ Hunting period (hunting vs. nonhunting [nonhunt.]).

At the home range scale, models including landscape covariates were also selected (Table 2). There was moderate to strong evidence on the age time scale and strong evidence on the annual time scale that the landscape variability described by PC1 affected survival. The PC1 characterizes a gradient from high‐human‐modification areas, including high forest land cover, to areas with more shrub and grass land covers farther from human settlements and roads (Appendix S17; H9 & H10). One model selected on the annual time scale provided weak to moderate evidence that if lynx used locations with on average higher habitat suitability, their survival probability would be higher (H11). Finally, one selected home range model showed weak evidence that lower human populations and urban cover could increase survival times. The evidence that landscape characteristics affected survival was stronger at the home range scale (i.e., higher posterior probabilities of estimates in the selected models, H7).

Considering the selected models that included frailty as a spatially correlated risk (GRF frailty), we found the effects posed by unobserved drivers of survival (i.e., those not explained by the covariates) were relatively restricted spatially (Table 3). This means that the combined effects of unexplained factors that caused variation in survival were similar for individuals found close together (e.g., approximately 5% lower correlation at 10 km). The similarity of these effects decreased rapidly with increasing distance between individuals (e.g., approximately 50% lower correlation at 150 km). There was also a relatively high variance in this component, indicating considerable heterogeneity in the unobserved factors (τ^2^ from 0.1 to 0.9).

The selected models were consistent with statistical modeling framework assumptions (Appendices S18–S21), and the coefficient estimates were stable for all covariates with weak to very strong evidence (Table 3; Appendix S11).

## DISCUSSION

Making use of a large telemetry data set, we provided the first continental‐scale assessment of survival rates and cumulative incidence of cause‐specific mortalities for a large carnivore in the human‐dominated landscapes of Europe. This offers managers and researchers the most up‐to‐date knowledge on lynx survival probability. We found that human‐related mortalities, including illegal killing, legal hunting, and vehicle collisions, exceeded natural mortality causes, as was previously shown in some studies focused on specific lynx populations (Andrén et al., 2006; Breitenmoser‐Würsten et al., 2007; Schmidt‐Posthaus et al., 2002; Sindičić et al., 2016). Illegal killing was the most important source of mortality risk for lynx in protected populations. In hunted populations, illegal killing posed a similar risk as legal hunting, and lynx were similarly likely to die naturally. Indeed, across our study sites, we found strong evidence that anthropogenic and hunting mortalities were only partially compensated by reductions in natural mortality or other causes. Based on multivariate survival models, we found lynx had an approximately 20% probability of reaching 15 years of age. Survival was mostly driven by sex, season, and landscape characteristics. For both landscape and home range scales, lynx that used areas with fewer anthropogenic influences farther from human infrastructure were likely to survive longer, provided unobservable local conditions, such as illegal killing, were not detrimental.

As we showed for lynx, illegal killing is a prominent concern for many large carnivore species (Carter et al., 2017). In this context, sustainable harvest is often seen as a method to balance contrasting stakeholder wishes in the political arena (Linnell et al., 2010). In our study areas, hunting mortality was at best only partially compensated by lower mortality due to other causes. This was also the case when all anthropogenic mortality causes were pooled. Natural mortality occurred at similar rates in hunted populations and was not low enough to compensate fully. Indeed, lower rates of all other mortality causes were also not sufficient. Therefore, it is likely that natural mortality rates are already at low levels due to high anthropogenic mortality rates across the lynx's European range. We found no evidence that hunting mortality compensates for illegal killing; therefore, our results do not support the claim that hunting quotas directly help reduce illegal killing mortality (e.g., Lindsey et al., 2007).

Hunting aims to improve societal tolerance of large carnivores (Treves, 2009) rather than to change individuals’ actions. Therefore, the coincidence of high illegal killing in hunted populations that we found is not necessarily unexpected. In Europe, illegal killing only appears to threaten small, reintroduced populations of lynx (Arlettaz et al., 2021; Heurich et al., 2018), whose small sizes necessitate conservation. Given the unclear relationship between illegal killing and hunting of lynx, introducing hunting management in protected populations would be highly contentious (Ghasemi, 2021). Although our results provide a first glimpse into the partially compensatory relationship between legal hunting and illegal killing, time series of tracking data in periods or areas with varying hunting quotas would be required to further disentangle them. However, these data are currently lacking. Our results imply that hunting needs to be managed carefully to ensure that the combination of natural mortality and the at least partially additive effect of hunting mortality does not exceed what the population's growth rate can tolerate. This requires effective monitoring and adaptive management feedback loops that adjust quotas (Cusack et al., 2022). Because illegal killing will likely persist, adaptive management plans, including feedback from careful monitoring, are necessary to react to dynamic situations (Andren et al., 2020). This would enable, for example, quotas to be reduced in hunted populations if illegal killing is discovered, or in hunted and protected populations, law enforcement resources could be diverted to problem areas as they emerge. Good enforcement is necessary for implementing environmental law, and in the case of large carnivores in Europe, there appears to be room for improvement (Arlettaz et al., 2021).

Roads can pose a high risk to wide‐ranging species like carnivores (Bastianelli et al., 2021; Grilo et al., 2015). Vehicle collisions had a relatively low incidence compared with other sources of mortality. This is likely due to relatively low road densities where lynx often occur (e.g., Niedziałkowska et al., 2006). However, we still found that lynx that used habitats farther from roads tended to survive longer. Roads can also be dangerous for carnivores because they provide access for hunters and likewise for illegal killing (e.g., Person & Russell, 2008). Despite this, vehicle collisions must be considered an important risk that is especially concerning for small and isolated populations (Taylor et al., 2002). Vehicle collisions presented a higher risk for younger individuals, with 50% of events occurring within 3 years of age. This inevitably reduces exchange among lynx populations in Central Europe because natal dispersal is a key process for connectivity (Kramer‐Schadt et al., 2004). This problem is also set to increase as road density and traffic intensity expand (Meijer et al., 2018). Improving green infrastructure and landscape permeability is a tangible intervention that may reduce lynx mortality and support population exchange. However, this depends on identifying vehicle collision hotspots for optimal placement of mitigation measures (e.g., Bil et al., 2019). Illegal killing is currently of greater magnitude and itself creates invisible barriers to lynx dispersal.

As with other large carnivores (Goodrich et al., 2008; Rauset et al., 2016), we found females had higher survival probabilities than males. This is consistent with the hypothesis that male life histories infer higher risks to secure more mates (Trivers, 1985) and is expected from sex‐biased movement and dispersal (Samelius et al., 2012), with its high associated fitness costs (Lucas et al., 1994). Conversely, female lynx have higher natal philopatry (Krojerová‐Prokešová et al., 2019) and appear to avoid risky behavior compared to males (Bunnefeld et al., 2006). In some hunted populations, males are at higher risk of being hunted than females because of female subquotas (Nilsen et al., 2012). In our results, male lynx in hunted populations had a higher risk of being hunted than females; however, their illegal killing rate was similar. If illegal kills occur by chance, through opportunistic encounters, poisoning, or snaring, one might expect a low sex bias, as observed. However, sexually dimorphic prey selection (e.g., Sunde & Kvam, 1997) could modulate lynx's spatial behavior and therefore risk for human‐caused mortality. Sex‐specific survival seems to be, therefore, the aggregation of sex‐specific factors and behavior with local prey communities and human factors, such as hunting quotas. Our data set was not sufficient to consider sex‐specific additive and compensatory mortality hypotheses, but these relationships could have important implications for management (e.g., Toïgo et al., 2008).

Solitary felids exhibit sex‐specific seasonal activities that could affect their survival (Sandell, 2019). As expected, lynx hunting season was associated with the highest mortality risk (Andrén et al., 2022). After the hunting periods, which occur during winter, we also found winter without hunting pressure to pose a high risk. Weather conditions are unlikely to affect survival because lynx are well adapted to hunting in snow (Nilsen et al., 2009) when prey is less mobile (Mech et al., 1987). Winter could be linked to higher intraspecific competition for mates (Mattisson et al., 2013). More importantly, during winter, wildlife also leave tracks in the snow that may increase their likelihood of opportunistic illegal killing (e.g., Santiago‐Ávila & Treves, 2022), especially in areas close to human activities, where detection is most likely and where prey may be aggregated (e.g., feeding sites or agricultural areas; Bunnefeld et al., 2006). Despite seasonality in lynx life history, there was little statistical evidence to differentiate between autumn (females with kittens become more mobile), spring (juveniles reach independence), and summer (kitten rearing).

Habitat selection takes place at various spatial and temporal scales to maximize fitness (Johnson, 1980). Typically, the most limiting factors for individual fitness are believed to be addressed at coarser scales (Rettie & Messier, 2000). Considering the high anthropogenic mortality rates of lynx, the hierarchical habitat selection of lynx seems consistent with the limiting factor hypothesis, wherein lynx avoid human disturbances more at the landscape scale than within the home range (Ripari et al., 2022). Habitats with little human disturbance are therefore considered an advantage for lynx occurrence (Oeser, Heurich, Kramer‐Schadt, Andrén et al., 2023) and might be expected to improve a lynx's chance of survival. Following this, we hypothesized that survival depends on the use of different factors at different spatial scales. We found lynx survived longer when their home range composition and within‐home‐range habitat use were associated with areas with little human modification and areas farther from human infrastructure, such as roads and settlements. The evidence of this was strongest regarding habitat use at the home range scale. These results were consistent with our hypothesis based on avoidance of human disturbances at the landscape scale (H8) but did not strongly reflect the hierarchical process expected (H7) in which lynx survival was correlated more with use of habitat associated with prey resources in the home range (H9) than with avoidance of the main mortality risk at the coarser scale. Further, we found weak to moderate evidence that use of more suitable habitat at the fine scale increased survival rates (H11).

Local studies, partly based on the same data as this study, showed disparate results. Lynx survival in Norway was shown to decrease with increasing accessibility of forests (Basille et al., 2013), whereas there was little effect of landscape on survival in neighboring Sweden (Andrén et al., 2022). In this analysis, across more study areas, lynx embedded in more human‐modified landscapes seem to be at higher risk at both fine and coarse scales. This suggests that not all lynx can minimize the fitness cost at the coarse scale and must also use habitats cautiously at the home range scale. In addition to landscape drivers, we found that some of the spatial variation and heterogeneity of risk was due to unobserved local factors. For instance, areas farther from human infrastructure may observably represent refuges; however, illegal activities can go undetected in more remote areas (Rauset et al., 2016), which would be an unobservable local risk factor. Survival is dependent on diverse local factors, which are partly difficult to describe with landscape proxies. For example, attitudes or actions opposing lynx can arise over competitive interest for game species (Treves, 2009) or livestock depredation (Abade et al., 2014). Furthermore, many processes that affect survival depend on con‐ and heterospecific population densities, including prey availability, competitive killing, or disease transmission (Brøseth et al., 2010; Murray et al., 1999; Palomares & Caro, 1999). Although some factors are ultimately conveyed by habitat use (e.g., individuals forced into less suitable areas due to high conspecific density), not all factors can be quantified, such as prey vulnerability, human activities, or cultural aspects of tolerance, and especially the behaviors of small groups of humans who engage in illegal killing. By statistically accounting for the variability of these confounding factors, we could understand the effect of habitat use alone. Our results thereby corroborate the prevailing wisdom that conserving habitat and preventing further fragmentation are fundamental ingredients for lynx conservation.

We showed that lynx survival was partly driven by landscape factors, particularly correlates of human influences. Habitats with little human disturbance and human infrastructure offer lynx the best survival chances, provided unobserved factors are not detrimental. The at best partially compensatory relationship between anthropogenic and other mortality causes highlights that Europe's lynx occurrences are at high risk of mortality. In fact, with natural mortality already at low levels in hunted and protected populations, mortality causes are likely close to additive already. For this reason, the persistence of small lynx populations should not be taken for granted. Whether lynx can thrive in novel patches will depend on dynamic local conditions, including societal perceptions, management actions (e.g., assisted dispersal), and ecological connectivity among patches (Jaroszewicz et al., 2021; Linnell et al., 2015). These factors should be considered in future research to better understand spatial variation in population dynamics of lynx.

## Supporting information



Supplementary Materials.
