# Supplementary material for: Survival of Eurasian lynx in the human‐dominated landscape of Europe
Source: Conserv Biol. 2025 Jan 14;39(3):e14439. doi: 10.1111/cobi.14439 (PMC12124177; doi:10.1111/cobi.14439)
Supplement: Supplementary file 1 — Supplementary Materials. [file COBI-39-e14439-s001.pdf]

Survival of Eurasian lynx occupying the human-dominated landscape of Europe

Appendices

Appendix S1 Summary characteristics of lynx populations represented with data in this study.

Population[a]	Lynx[b] [f/m]	Lynx-years	Deaths	Area[c] (km^2)	Country	Management[d]
1. Scandinavian	418 [206/212]	717.51	159	767	Norway	Hunting (Feb 1 – Mar 31)[e]
					Sweden	Hunting (1995-2006: Jan 1 – Apr 15, 2007-pres.: Mar 1 – Mar 31)
3. Baltic[f]	55 [19/36]	53.58	10	326	Estonia	Hunting (2003-pres.: Dec 1 – Feb 28/29)
					Latvia	Hunting (2004-2019: Dec 1 – Mar 31, 2020-2022.: Jan 1 – Mar 31)
					Poland	Protected
4. Carpathian	22 [5/17]	26.55	3	447	Czechia	Protected
					Poland	Protected
					Slovakia	Protected
5. Balkan	12 [5/7]	11.22	0	266	N. Macedonia	Protected
6. Dinaric-Southeastern Alpine	56 [17/39]	51.17	5	331	Croatia	Protected
					Italy	Protected
					Slovenia	Protected
7. Bohemian-Bavarian-Austrian	20 [7/13]	30.27	5	184	Czechia	Protected
					Germany	Protected
8. Alpine	47 [28,19]	68.99	17	153	Austria	Protected
					Switzerland	Protected
9. Jura Mountains	38 [24/14]	77.05	17	259	France	Protected
					Switzerland	Protected
11. Harz Mountains	9 [3/6]	11.56	3	340	Germany	Protected

Baden-Württemberg (nonpermanent occurrence)	4 [0/4]	3.42	0	285	Germany	Protected

[a]Numbering after Chapron et al. (2014).
[b]Individuals were captured using box traps, foot snares, or darted from a helicopter, and equipped with VHF (432 deployments) or GPS (442 deployments) collars.
[c]Utilized area calculated as a sum of seasonal utilization areas/sum of tracking years/n lynxes.
[d]Where stated, management refers to the legal hunting season of lynx, otherwise no legal hunting present (except for exceptional circumstances).
[e]Radio-collared lynx from Norway's Hedmark region were not hunted in part of the study period, and therefore this period (total 71.60 lynx years) was not included in hunted or protected groupings for nonparametric estimates or the survival modelling.
[f]Including Białowieża, Poland.

Appendix S2 Animal capture approval information.

Study area; period; responsible party	Details
Bavarian Forest, Germany; 2004-2012; Marco Heurich, Bavarian Forest National Park	The research program in the Bavarian Forest is managed by the Administration of the Bavarian Forest National Park. Lynx captures were conducted in accordance with European and German animal welfare laws. Animal captures and experimental procedures were approved by the Ethics Committee of the Government of Upper Bavaria and fulfils their ethical requirements for research on wild animals (Reference number 55.2-1-54-2531-82-10).
Bohemian Forest, Czechia; 1997-2012; Elisa Belotti and Ludek Bufka, Sumava National Park, Czechia	Lynx were captured following established standard protocols (described in: Belotti E, Weder N, Bufka L, Kaldhusdal A, Küchenhoff H, Seibold H, et al. (2015) doi:10.1371/journal.pone.0138139). The handling protocol was approved by the Czech Central Commission for Animal Welfare and fulfils their ethical requirements for research on wild animals (permit number: 55.2-1-54-2532-82-10). In addition, permits for wild animal capture were obtained from the Czech Central Commission for Animal Welfare (permit numbers: 44048/2008-17210, 44048/2008-10001) and the Czech Ministry of Environment (permit number: 41584/ENV/10-1643/620/10-PP8).
Moravian Karst, Czechia; 2018-2019; Martin Duľa, Department of Forest Ecology, Mendel University, Brno	The research program in the Moravian Karst was managed by the Mendel University in Brno. Lynx captures were conducted in accordance with European and Czech animal welfare laws. Permits for animal capture and handling were obtained from the PLA Moravian Karst Administration and the Ministry of Environment of the Czech Republic, permit numbers: SR/0081/JM/2017; 34128/ENV/17-2146/630/17.
PLA Beskydy, Western Carpathians, Czechia; 2020-ongoing; Martin Duľa, Department of Forest Ecology, Mendel University, Brno	The research program in PLA Beskydy is managed by the Mendel University in Brno. Lynx captures were conducted in accordance with European and Czech animal welfare laws. Permits for animal capture and handling were obtained from the PLA Beskydy Administration and the Ministry of Environment of the Czech Republic, permit numbers: SR/0031/BE/2019; MZP/2020/630/167
PLA Beskydy, Western Carpathians, Czechia; 2011-2015; Jarmila Krojerová, Institute of Vertebrate Biology of the Czech Academy of Sciences, Brno	The research program in PLA Beskydy was managed by the Institute of Vertebrate Biology of the Czech Academy of Sciences in Brno. Lynx captures were conducted in accordance with European and Czech animal welfare laws. Permissions for lynx captures were issued by the Protected Landscape Area Administration Beskydy (no. 6535/BE/2008) and approved by the Ethics Committee of the Czech Academy of Sciences (no. 130/2010).
Białowieża Promeval Forest, Poland; 1991-1996, 2004-2012; Krzysztof Schmidt, Mammal Research Institute Polish Academy of Sciences, Poland	Institute PAS in Białowieża, Poland. Lynx captures were conducted in accordance with European and Polish animal welfare laws. Permissions for lynx trapping were issued by the Ministry of Environment and the National Ethics Committee for Animal Experiments (no. DB/KKE/PL—110/2001) and the Local Ethics Committee for Animal Experiments at the Medical University of Białystok, Poland (no. 52/2007).

Croatia; 2000 – ongoing; Magda Sindičić, Faculty of Veterinary Medicine University of Zagreb, Croatia	The research program in Croatia is managed by the Faculty of Veterinary Medicine University of Zagreb. Lynx captures were conducted in accordance with European and Croatian animal welfare laws. Animal captures and experimental procedures were approved by the Ministry for Nature protection and the permit is renewed each year.
Mavrovo NP and surroundings in western Macedonia; 2010 – ongoing; Dime Melovski, Macedonian Ecological Society, North Macedonia	The research programme in Macedonia is managed by the Macedonian Ecological Society. Trapping of the Balkan lynx was approved by the Macedonian Ministry of Environment and Physical Planning (permits number: 11-2186/2; 11-546/2;11-1006/10).
Carpathians, Slovakia; 2018-2021; Jakub Kubala and Branislav Tám, National Zoo Bojnice, Slovakia	The research program in the Slovak Carpathians is managed by the Technical university in Zvolen, National Zoo Bojnice and Diana - Carpathian Wildlife research, in accordance with ARRIVE guidelines. All procedures were approved by the Ministry of Environment of the Slovak Republic (permits no. 7402/2013-2.2; 366/2016-2.3; 6933/2019-9.2 [7/19 – rozkl.]). No mortalities occurred during or after captures and no complications were observed due to collaring.
Dinaric Mts. and Julian Alps, Slovenia; 2006 – ongoing; Miha Krofel, University of Ljubljana, Biotechnical faculty, Slovenia	The research program in Slovenia is managed by the University of Ljubljana. Lynx captures were conducted in accordance with European and Slovenian animal welfare laws. Animal captures and experimental procedures were approved by the committee at the Slovenian Environmental Agency, who issued the research permits (no. 35601-45/2006-6 and 35601-76-2020-6).
Dinaric Mts. and Julian Alps, Slovenia; 2006 – ongoing; Nives Pagon and Rok Černe, Slovenia Forest Service	Lynx captures and translocations were conducted in accordance with European and Slovenian animal welfare laws. Animal captures and translocations, as well as experimental procedures were approved by the Slovenian Environmental Agency - Ministry of Environment and Spatial Planning, which issued permits No. 35601-29/2018-4 and 35601-90/2018-4.
Norway; 1995-2017; John Odden et al., Norwegian Institute for Nature Research	The Norwegian part of the Scandinavian research project, SCANDLYNX, is managed by Norwegian Institute for Nature Research (NINA). All capture and handling procedures were approved by the Norwegian Experimental Animal Ethics Committee and followed their ethical requirements for research on wild animals (permit numbers (FOTS ID 2827, FOTS ID1391, 13912012/206992, 2010/161554, 2010/161563, 08/127430, 07/81885, 07/7883, 2004/48647, 201/01/641.5/FHB127/03/641.5/fhb, 1460/99/641.5/FBe, 1081/97/641.5/FBe, and NINA 1/95). In addition, permits to capture wild animals were provided by the Norwegian Environment Agency.
Latvia; 2007-2008; Guna Bagrade, Latvian State Forest Research Institute	The research program was managed by the Latvian State Forest Research Institute "Silava". Lynx captures were conducted in accordance with European and Latvian animal welfare laws. The study was designed to minimize animal stress and handling time, and to ensure animal welfare, as defined in the guidelines for the ethical use of animals in research. Permissions for lynx trapping were issued by headquarter of the State Forest Service (SFS) (permit No. 1-10/225 and No. 16-9/318). In addition, trapping process was approved by local authority of the SFS (No. 08-2/333).

Baden-Wuerttemberg, Germany; 2015 – ongoing; Micha Herdtfelder, Forest Research Institute Baden-Wuerttemberg	The research program in Baden-Wuerttemberg is managed by the Administration of the Forest Research Institute Baden-Wuerttemberg. Lynx captures are conducted in accordance with European and German animal welfare laws. Animal captures and experimental procedures were approved by the Ethics Committee of the governmental district of Freiburg and fulfils their ethical requirements for research on wild animals.
Sweden; 1993 – ongoing; Henrik Andrén, Jens Persson, et al., Grimsö Wildlife Reseearch Station, SLU	The Swedish part of the Scandinavian research project, SCANDLYNX, is managed by Grimsö Wildlife Research Station (SLU). All capture and handling procedures were approved by the Swedish Animal Ethics Committee and followed their ethical requirements for research on wild animals (permit C275/95, C16/0). In addition, permits to capture wild animals were provided by the Swedish Environment Protection Agency (permit NV-07775-16).
Swiss Jura, Switzerland; 1988-1998; Carnivore ecology and wildlife management (KORA)	Lynx were captured following established standard protocols (described in Breitenmoser & Haller, 1993) and with all permits required according to Swiss legislation for capturing, immobilizing, and radio tagging lynx (capture permits from Hunting and Fishing Administration of the canton of Solothurn issued on 27.01.1988, the Cantonal Inspectorate of Fishing and Hunting of the canton of Neuchâtel issued on 13.06.1988 until 31.12.1988, and Forestry and Wildlife Service of the canton of Vaud issued 14.07.1988, capture permits from the Federal Office for the Environment: issued on 6.07.1994 until 31.12.1998; animal experimentation permit from the supervisory committee for animal experiments of the Cantonal Veterinary Service and Galli-Valerio institute of the canton of Vaud: 512 and 1047; animal experimentation permit from the veterinarian of the canton of Jura: 2/02).
French Jura, France; 1995-1998; Nolwenn Drouet-Hoguet, French Office for Biodiversity, Vincennes, France	The research program in France was managed by the former National Office for Hunting and Wildlife (ONCFS), now merged with the French Office for Biodiversity (OFB). Lynx captures were conducted in accordance with European and French animal welfare laws. Animal captures and experimental procedures were approved and licensed by the appropriate authorities. The research was conducted in cooperation with the Ministry of Environment, Nature and Landscapes Directorate.
North-western Alps, Switzerland; 1997-2001; Carnivore ecology and wildlife management (KORA)	Lynx were captured following established standard protocols (described in Breitenmoser & Haller, 1993; Ryser et al., 2005; Ryser-Degiorgis et al., 2002; Zimmermann, Breitenmoser-Würsten & Breitenmoser, 2005) and with all permits required according to Swiss legislation for capturing, immobilizing, and radio tagging lynx (capture permits from the Federal Office for the Environment: issued on 27.12.1996 until 31.12.2001; animal experimentation permit from the Animal Welfare Commission of the Office for Agriculture of the Canton of Bern: 66/97 and 8/00; animal experimentation permit from the supervisory committee for animal experiments of the cantonal veterinary service of the Canton of Vaud: 1047.1).

Harz Mountains, Germany; 2008 – 2017; Ole Anders and Tomma Lilli Middelhoff, Harz National Park, Germany	The research program was managed by the Harz National Park Administration. The capture, anesthesia and collaring of lynxes took place in accordance with all relevant animal welfare regulations and on the basis of the following animal experiment approvals by the Lower Saxony State Office for Consumer Protection and Food Safety (Niedersächsisches Landesamt für Verbraucherschutz und Lebensmittelsicherheit, Oldenburg, Germany): File number: 33.11.42502-04-082/07 (accepted by the Free State of Thuringia, FN: 22-2684-04-015-001/09), FN: 33.14-42502-04-10/0201 (accepted by the Free State of Thuringia, FN: 22-2684-04-015-001/10), FN: 33.19-42502-04-14/1571 (accepted by the State of Saxony Anhalt, FN: 42502-2-1289 Harz), FN: 33.19-42502-04-19/3229 (accepted by the State of Saxony Anhalt, FN: 42502-2-1614 Harz)
Kalkalpen, Austria; 2011-2015; Christian Fuxjäger, Nationalpark O.ö. Kalkalpen, Molln, Austria	The lynx species conservation project in the Kalkalpen NP is carried out in close cooperation with the Upper Austrian Nature Conservation Department. The necessary licences for lynx captures and tagging were obtained from the province of Upper Austria.

Appendix S3 Summary of illegal kill criteria of Andrén et al. (2006)

Detecting illegal killing from radio-telemetry data can be difficult because the transmitters are often destroyed by the perpetrators, hence many lynx simply vanish (i.e. stop transmitting location data). Lynx can also stop transmitting location data for other reasons, especially due to technical failure of the equipment (Hofman et al. 2019). It is therefore important to use a set of transparent criteria for deciding which cases to include as illegal kills and which to exclude (censored with unknown fate). To classify disappearances as illegal kills, we followed the criteria set out by Andrén et al. (2006).

If either of criteria 1 or 2 was met:

1. Radio-transmitter was found in strange places, e.g., in a lake and the collar had been cut off, or else a collar was found smashed.
2. Sudden disappearance of two separate radio-transmitters on one individual (i.e., GPS collar and VHF implant) simultaneously, despite careful tracking after disappearance.

To distinguish particularly from dispersal events or transmitter failure, if criteria 3 and 4 were met:

3. Illegal killing suspected if a resident adult disappeared suddenly despite careful tracking (i.e., dispersal unlikely).
4. No indication of technical problems with the radio-transmitter:
    a. No strange or weak signals.
    b. Over half of the battery life still available.
    c. Area surveyed carefully for signals.

To distinguish particularly for juvenile or subadult lynx without their own established home-range and in dispersal phase, if the criteria 5 through 7 were met with criteria 4:

5. If the individual disappeared with a new radio-transmitter.
6. The dispersal had been carefully followed, i.e., known dispersal direction.
7. Careful radio-tracking immediately after the disappearance (i.e., searching area for VHF signal).

In these cases, disappearances were classified with the fate illegal killing. Else, if none of the criteria was met, the lynx fate was regarded as unknown, and the end of the tracking period was censored (no mortality).

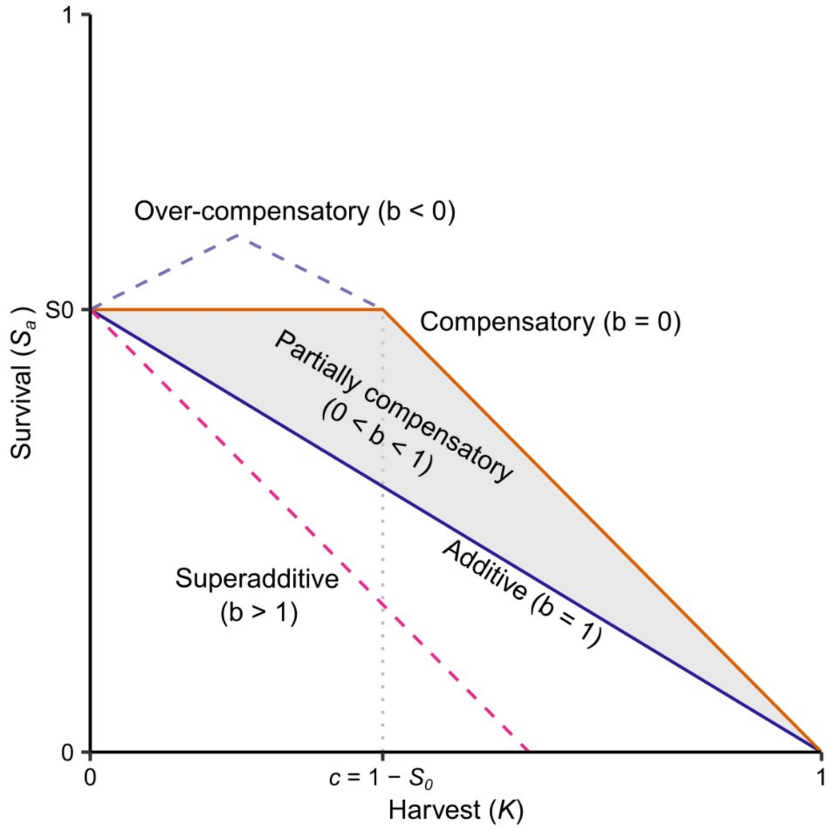

Appendix S4 Graphical representation of the relationship between annual survival (S_a) and harvest rate (K), following Sandercock et al. (2011) who described by the formula $S_a = S_0 (1 - bK)$, where S_0 is the baseline survival rate without hunting management, b is the negative slope coefficient (i.e., slope coefficient b is the slope multiplied by -1), and c = 1 – S_0, the threshold where the no more compensation is possible. Three hypotheses are labelled: 1) Additive (b = 1, negative trend) where survival declines with harvest (below lower dashed line), 2) compensatory (b = 0, no trend) where survival is not affected by harvest (upper dashed line), and 3) partially compensatory (0 < b < 1, negative trend with slope > -S_0) which lies between hypotheses 1) and 2) (grey region). For the additive line with intercept at S_0, b = -S_0 in other words the slope coefficient b is the slope multiplied by -1.

Appendix S5 Summary and descriptions of parameters used in survival modelling.

Type	Parameter	Description	Time-varying	Resolution	PCA[a] covariate	Note/data source[b]
Survival object (response)[a]	Start time	Time of first observation/left truncation time.		Day		Start of tracking period or observation split (season, year).
	Stop time	Time of last observation/event time/censoring time.		Day		End of tracking period or observation split (season, year).
	Event	Death event/censoring.				Cause-specific deaths used in competing risk analysis.
Individual	Subject number	Unique individual ID.	Yes			Used for IID frailties and grouping observations with time-varying covariates.
	Sex	Female, male.	Yes			
	Age/age class	Juvenile (age < 1), subadult (1 ≤ age < 2), adult (age ≥ 2).	No			Annually recurrent time scale only (nonparametric estimates).
	Location	Coordinates (lon, lat).	No			Raw locations used in area delineation, centroids used in GRF frailty.
Aggregate	Habitat suitability index	Lynx habitat suitability index, 'global' model predictions of scale-integrated habitat selection (multiplication of home range and within home range selection scales).	Yes	100 m	No	Multi-scale global model habitat suitability index of Oeser et al. (2023).
Natural	Season (age time scale only)	Spring (mar-may), summer (jun-aug), autumn (sep-nov), winter (dec-feb), hunting (country specific).	Yes	Days.	No	Defined *a priori* by management regimes (Appendix S1).
	Hunting period (annual time scale only)	Hunting, nonhunting (country specific).	Yes	Days.	No	Defined *a priori* by management regimes (Appendix S1).
	Ruggedness	Index of elevational variability based on a digital elevation model.	Yes	100 m	Yes	Shuttle Radar Topography Mission (Farr et al., 2007)
	Forest integrity	Index of forest naturalness and intactness.	Yes	300 m	Yes	Grantham et al. (2020)
	Greenness variability	Landsat surface reflectance metric.	Yes	100 m, annual	Yes	Oeser et al. (2020)
Human	Human modification index	Metric that reflects the amount the landscape	Yes	1 km	Yes	Kennedy et al. (2019)

		is modified by 13 human stressors. Including: settlements, agriculture, transportation, and other infrastructures.				
	Accessibility	Travel time to cities in minutes.	Yes	1 km	Yes	Weiss et al. (2018)
	Distance to major roads	Minimum distance to motorway, trunk, and primary roads.	Yes	100 m	Yes	Derived from OpenStreetMap Contributors ® data, downloaded from https://download.geofabrik.de
	Distance to minor roads	Minimum distance to secondary, tertiary, and unclassified roads.	Yes	100 m	Yes	Derived from OpenStreetMap Contributors® data, downloaded from https://download.geofabrik.de
	Distance to settlements	Minimum distance to urban land cover.	Yes	100 m	Yes	Derived from Buchhorn et al. (2020)
	Human population density	Persons per pixel in 2015.	Yes	250 m	Yes	Freire et al. (2016)
	Management	Legal hunting, no legal hunting (see Appendix S1)[d].	Yes	Categorical by country.	No	Included in the time-varying categorical variable 'season' (age time scale) and 'hunting period' (annual time scale).
Landcover	Forest	Fractional cover of forest.	Yes	100 m, annual	Yes	Buchhorn et al. (2020)
	Shrub	Fractional cover of shrub.	Yes	100 m, annual	Yes	Buchhorn et al. (2020)
	Crop	Fractional cover of cropland.	Yes	100 m, annual	Yes	Buchhorn et al. (2020)
	Grass	Fractional cover of grassland.	Yes	100 m, annual	Yes	Buchhorn et al. (2020)
	Urban	Fractional cover of urban.	Yes	100 m, annual	Yes	Buchhorn et al. (2020)

[a]Response parameters also refer to nonparametric survival and cumulative incidence of cause-specific mortality estimates.

[b]PCA, principal component analysis.

[c]Abbreviations: IID, independent and identically distributed frailty; GRF, Gaussian random field frailty.

[d]Lynx from the Norwegian Hedmark region were not subject to normal hunting pressure, therefore those individuals had no hunting season and were removed from survival models.

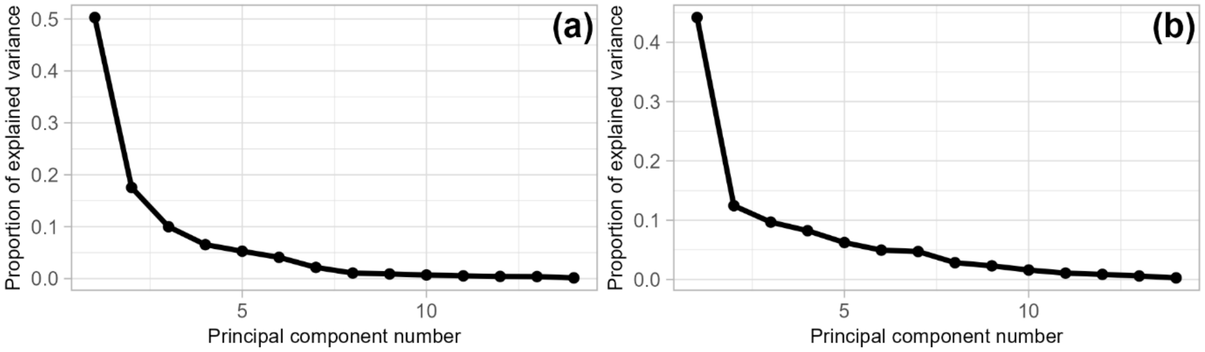

Appendix S6 Scree plots indicating the proportion of variance explained by each principal component used in the a) landscape and b) home-range multivariate survival models. In both cases only the first 5 (variance explained > 5%) principal components were considered (PC biplots Appendices S16 and S17).

Appendix S7 Conditional survival and cumulative incidence of cause-specific mortalities calculated on the age time scale, for all lynx and grouped by management types.

Group	Time (age)	N_R[a]	Conditional survival probability[b]		Cause-specific mortality cumulative incidence[c]									
					Natural		Hunting		Illegal kill		Vehicle collision		Unknown	
			N_E[d]	Est. (CI)[e]	N_E	Est. (CI)	N_E	Est. (CI)	N_E	Est. (CI)	N_E	Est. (CI)	N_E	Est. (CI)
All populations	1	181	22	0.78 (0.68,0.86)	8	0.077 (0.019, 0.131)	1	0.009(0, 0.026)	1	0.006(0, 0.016)	2	0.022(0, 0.056)	10	0.123(0.047, 0.193)
	2	190	34	0.8 (0.73,0.85)	10	0.135 (0.069, 0.196)	5	0.04(0.008, 0.072)	11	0.079(0.035, 0.121)	5	0.056(0.011, 0.099)	3	0.141(0.063, 0.212)
	3	259	44	0.77 (0.71,0.83)	9	0.176 (0.108, 0.24)	16	0.128(0.076, 0.177)	14	0.154(0.098, 0.207)	2	0.068(0.02, 0.113)	3	0.156(0.077, 0.227)
	4	158	42	0.8 (0.74,0.85)	9	0.214 (0.144, 0.279)	10	0.179(0.12, 0.233)	17	0.223(0.161, 0.28)	2	0.078(0.029, 0.124)	4	0.173(0.094, 0.245)
	5	107	27	0.8 (0.72,0.86)	2	0.229 (0.157, 0.295)	14	0.275(0.203, 0.34)	7	0.264(0.197, 0.325)	2	0.095(0.041, 0.146)	2	0.186(0.106, 0.259)
	6	79	11	0.88 (0.79,0.93)	0[f]	0.229 (0.157, 0.295)	1	0.284(0.21, 0.351)	8	0.329(0.252, 0.398)	1	0.105(0.048, 0.159)	1	0.195(0.114, 0.269)
	7	61	12	0.83 (0.73,0.9)	2	0.253 (0.175, 0.324)	6	0.349(0.263, 0.426)	2	0.348(0.268, 0.419)	0	0.105(0.048, 0.159)	2	0.217(0.131, 0.294)
	8	43	12	0.77 (0.63,0.86)	0	0.253 (0.175, 0.324)	3	0.393(0.297, 0.476)	7	0.437(0.34, 0.52)	0	0.105(0.048, 0.159)	2	0.254(0.156, 0.341)
	9	26	5	0.86 (0.7,0.94)	1	0.279 (0.186, 0.362)	1	0.413(0.31, 0.5)	3	0.479(0.375, 0.566)	0	0.105(0.048, 0.159)	0	0.254(0.156, 0.341)
	10	19	4	0.82 (0.6,0.93)	1	0.312 (0.199, 0.409)	0	0.413(0.31, 0.5)	2	0.528(0.407, 0.624)	1	0.148(0.045, 0.24)	0	0.254(0.156, 0.341)
	11	15	1	0.93 (0.59,0.99)	1	0.361 (0.211, 0.482)	0	0.413(0.31, 0.5)	0	0.528(0.407, 0.624)	0	0.148(0.045, 0.24)	0	0.254(0.156, 0.341)
	12	10	1	0.92 (0.54,0.99)	0	0.361 (0.211, 0.482)	0	0.413(0.31, 0.5)	1	0.567(0.425, 0.674)	0	0.148(0.045, 0.24)	0	0.254(0.156, 0.341)
	13	7	1	0.88 (0.39,0.98)	1	0.441 (0.218, 0.6)	0	0.413(0.31, 0.5)	0	0.567(0.425, 0.674)	0	0.148(0.045, 0.24)	0	0.254(0.156, 0.341)
	14	7	0	1 (1,1)	0	0.441 (0.218, 0.6)	0	0.413(0.31, 0.5)	0	0.567(0.425, 0.674)	0	0.148(0.045, 0.24)	0	0.254(0.156, 0.341)
	15	1	1	0.86 (0.33,0.98)	0	0.441 (0.218, 0.6)	0	0.413(0.31, 0.5)	1	0.629(0.438, 0.755)	0	0.148(0.045, 0.24)	0	0.254(0.156, 0.341)
Hunted populations	1	113	16	0.79 (0.68,0.87)	3	0.035 (0, 0.075)	1	0.012(0, 0.035)	1	0.009(0, 0.026)	2	0.028(0, 0.068)	9	0.137(0.049, 0.218)
	2	131	17	0.84 (0.76,0.9)	4	0.07 (0.017, 0.121)	4	0.05(0.006, 0.092)	5	0.058(0.012, 0.103)	2	0.049(0, 0.097)	2	0.156(0.065, 0.238)

	3	184	31	0.77 (0.69,0.84)	6	0.112 (0.05, 0.17)	16	0.168(0.099, 0.232)	7	0.115(0.054, 0.173)	1	0.059(0.005, 0.109)	1	0.162(0.071, 0.245)
	4	106	32	0.78 (0.71,0.84)	6	0.152 (0.084, 0.215)	10	0.237(0.16, 0.307)	12	0.189(0.118, 0.254)	1	0.065(0.01, 0.117)	3	0.179(0.087, 0.261)
	5	62	21	0.76 (0.66,0.84)	1	0.165 (0.093, 0.231)	13	0.356(0.264, 0.437)	3	0.218(0.141, 0.288)	2	0.092(0.026, 0.152)	2	0.199(0.104, 0.283)
	6	45	9	0.83 (0.7,0.91)	0	0.165 (0.093, 0.231)	1	0.371(0.276, 0.453)	7	0.322(0.219, 0.412)	1	0.11(0.036, 0.179)	0	0.199(0.104, 0.283)
	7	32	8	0.8 (0.65,0.9)	1	0.19 (0.103, 0.269)	5	0.454(0.341, 0.547)	1	0.338(0.232, 0.43)	0	0.11(0.036, 0.179)	1	0.217(0.117, 0.306)
	8	25	6	0.81 (0.62,0.91)	0	0.19 (0.103, 0.269)	3	0.511(0.387, 0.61)	3	0.406(0.279, 0.51)	0	0.11(0.036, 0.179)	0	0.217(0.117, 0.306)
	9	16	4	0.82 (0.59,0.93)	1	0.235 (0.11, 0.343)	1	0.535(0.405, 0.637)	2	0.455(0.316, 0.566)	0	0.11(0.036, 0.179)	0	0.217(0.117, 0.306)
	10	12	4	0.74 (0.45,0.89)	1	0.29 (0.124, 0.424)	0	0.535(0.405, 0.637)	2	0.533(0.362, 0.658)	1	0.174(0.025, 0.3)	0	0.217(0.117, 0.306)
	11	7	1	0.88 (0.39,0.98)	1	0.379 (0.131, 0.556)	0	0.535(0.405, 0.637)	0	0.533(0.362, 0.658)	0	0.174(0.025, 0.3)	0	0.217(0.117, 0.306)
	12	5	0	1 (1,1)	0	0.379 (0.131, 0.556)	0	0.535(0.405, 0.637)	0	0.533(0.362, 0.658)	0	0.174(0.025, 0.3)	0	0.217(0.117, 0.306)
	13	5	0	1 (1,1)	0	0.379 (0.131, 0.556)	0	0.535(0.405, 0.637)	0	0.533(0.362, 0.658)	0	0.174(0.025, 0.3)	0	0.217(0.117, 0.306)
	14	4	0	1 (1,1)	0	0.379 (0.131, 0.556)	0	0.535(0.405, 0.637)	0	0.533(0.362, 0.658)	0	0.174(0.025, 0.3)	0	0.217(0.117, 0.306)
Protected populations	1	51	4	0.85 (0.61,0.95)	3	0.099 (0, 0.215)	0	0(0, 0)	0	0(0, 0)	0	0(0, 0)	1	0.062(0, 0.174)
	2	53	12	0.75 (0.6,0.85)	5	0.2 (0.049, 0.327)	0	0(0, 0)	4	0.098(0.002, 0.186)	2	0.048(0, 0.111)	1	0.084(0, 0.2)
	3	70	12	0.78 (0.65,0.87)	3	0.246 (0.092, 0.373)	0	0(0, 0)	6	0.209(0.084, 0.317)	1	0.067(0, 0.139)	2	0.123(0, 0.243)
	4	46	10	0.83 (0.7,0.9)	3	0.289 (0.133, 0.416)	0	0(0, 0)	5	0.278(0.146, 0.389)	1	0.086(0.001, 0.163)	1	0.143(0, 0.265)
	5	40	5	0.87 (0.71,0.94)	1	0.307 (0.15, 0.435)	0	0(0, 0)	4	0.356(0.211, 0.474)	0	0.086(0.001, 0.163)	0	0.143(0, 0.265)
	6	32	2	0.95 (0.8,0.99)	0	0.307 (0.15, 0.435)	0	0(0, 0)	1	0.372(0.226, 0.49)	0	0.086(0.001, 0.163)	1	0.167(0.019, 0.293)
	7	28	4	0.87 (0.68,0.95)	1	0.333 (0.171, 0.464)	1	0.031(0, 0.09)	1	0.397(0.246, 0.517)	0	0.086(0.001, 0.163)	1	0.194(0.039, 0.324)
	8	17	6	0.7 (0.45,0.86)	0	0.333 (0.171, 0.464)	0	0.031(0, 0.09)	4	0.51(0.337, 0.639)	0	0.086(0.001, 0.163)	2	0.302(0.089, 0.464)
	9	9	1	0.92 (0.57,0.99)	0	0.333 (0.171, 0.464)	0	0.031(0, 0.09)	1	0.548(0.364, 0.679)	0	0.086(0.001, 0.163)	0	0.302(0.089, 0.464)

10	7	0	1 (1,1)	0	0.333 (0.171, 0.464)	0	0.031(0, 0.09)	0	0.548(0.364, 0.679)	0	0.086(0.001, 0.163)	0	0.302(0.089, 0.464)
11	8	0	1 (1,1)	0	0.333 (0.171, 0.464)	0	0.031(0, 0.09)	0	0.548(0.364, 0.679)	0	0.086(0.001, 0.163)	0	0.302(0.089, 0.464)
12	5	1	0.86 (0.33,0.98)	0	0.333 (0.171, 0.464)	0	0.031(0, 0.09)	1	0.613(0.388, 0.755)	0	0.086(0.001, 0.163)	0	0.302(0.089, 0.464)
13	2	1	0.67 (0.05,0.95)	1	0.556 (0, 0.806)	0	0.031(0, 0.09)	0	0.613(0.388, 0.755)	0	0.086(0.001, 0.163)	0	0.302(0.089, 0.464)
14	3	0	1 (1,1)	0	0.556 (0, 0.806)	0	0.031(0, 0.09)	0	0.613(0.388, 0.755)	0	0.086(0.001, 0.163)	0	0.302(0.089, 0.464)
15	1	0	1 (1,1)	0	0.556 (0, 0.806)	0	0.031(0, 0.09)	0	0.613(0.388, 0.755)	0	0.086(0.001, 0.163)	0	0.302(0.089, 0.464)

[a]Number at risk
[b]Calculated with product-limit (i.e., Kaplan-Meier) estimator. Survival probability at age T is conditional on survival until $T-1$.
[c]Calculated with weighted product-limit estimator. Cumulative incidence at time T is the probability of an event occurring conditional on survival until T.
[d]Number of events.
[e]Parameter estimate and 95% confidence interval.
[f]Caution should be taken when interpreting estimates with low N_E and N_R, as seen in wide CI.

Appendix S8 Annual survival and cumulative incidence of cause-specific mortalities calculated on the recurrent annual time scale, for all lynx and grouped by populations and management types.

Group	Age class[a]	N_R[b]	Conditional survival probability[c]		Cause-specific mortality cumulative incidence[d]									
					Natural		Hunting		Illegal kill		Vehicle collision		Unknown	
			N_E[e]	Est. (CI)[f]	N_E	Est. (CI)	N_E	Est. (CI)	N_E	Est. (CI)	N_E	Est. (CI)	N_E	Est. (CI)
All	subadult	181	34	0.8 (0.73, 0.85)	10	0.063 (0.024, 0.1)	5	0.032 (0.004, 0.059)	11	0.073 (0.031, 0.114)	5	0.034 (0.004, 0.063)	3	0.021 (0, 0.043)
	adult	931	163	0.81 (0.79, 0.84)	27	0.034 (0.021, 0.046)	51	0.067 (0.049, 0.084)	62	0.074 (0.056, 0.091)	9	0.012 (0.004, 0.019)	14	0.017 (0.008, 0.025)
Hunted populations	subadult	119	17	0.84 (0.76, 0.9)	4	0.037 (0.001, 0.071)	4	0.039 (0.001, 0.075)	5	0.05 (0.006, 0.092)	2	0.022 (0, 0.051)	2	0.021 (0, 0.05)
	adult	594	117	0.8 (0.76, 0.83)	17	0.033 (0.017, 0.048)	49	0.096 (0.07, 0.121)	38	0.07 (0.048, 0.091)	6	0.012 (0.002, 0.022)	7	0.012 (0.003, 0.021)
Hunted populations, male	subadult	57	13	0.73 (0.58, 0.84)	4	0.083 (0, 0.16)	3	0.066 (0, 0.137)	2	0.053 (0, 0.122)	2	0.051 (0, 0.118)	2	0.051 (0, 0.117)
	adult	252	60	0.76 (0.7, 0.8)	6	0.027 (0.005, 0.048)	33	0.15 (0.102, 0.196)	14	0.06 (0.029, 0.09)	3	0.014 (0, 0.03)	4	0.017 (0, 0.033)
Hunted populations, female	subadult	62	4	0.93 (0.83, 0.97)	0	-	1	0.018 (0, 0.052)	3	0.05 (0, 0.105)	0	0 (0, 0)	0	-
	adult	342	57	0.82 (0.78, 0.86)	11	0.037 (0.015, 0.059)	16	0.055 (0.028, 0.08)	24	0.077 (0.047, 0.106)	3	0.011 (0, 0.023)	3	0.009 (0, 0.019)
Protected populations	subadult	45	12	0.75 (0.6, 0.85)	5	0.111 (0.014, 0.198)	0	-	4	0.098 (0.002, 0.186)	2	0.048 (0, 0.111)	1	0.023 (0, 0.066)
	adult	308	44	0.84 (0.8, 0.88)	10	0.038 (0.014, 0.061)	1	0.004 (0, 0.011)	23	0.086 (0.051, 0.119)	3	0.012 (0, 0.025)	7	0.026 (0.007, 0.046)
Protected populations, male	subadult	24	5	0.78 (0.55, 0.9)	3	0.122 (0, 0.243)	0	-	2	0.108 (0, 0.24)	0	-	0	-
	adult	166	26	0.83 (0.76, 0.88)	6	0.043 (0.009, 0.077)	1	0.007 (0, 0.02)	13	0.089 (0.041, 0.134)	1	0.006 (0, 0.019)	5	0.036 (0.005, 0.067)
Protected populations, female	subadult	21	7	0.72 (0.5, 0.85)	2	0.098 (0, 0.217)	0	-	2	0.091 (0, 0.203)	2	0.085 (0, 0.192)	1	0.05 (0, 0.141)

	adult	142	18	0.86 (0.79, 0.91)	4	0.031 (0.001, 0.06)	0	-	10	0.081 (0.031, 0.129)	2	0.018 (0, 0.042)	2	0.015 (0, 0.036)
Reintroduced populations	subadult	37	10	0.75 (0.58, 0.86)	5	0.13 (0.017, 0.231)	0	-	3	0.088 (0, 0.179)	0	-	2	0.055 (0, 0.128)
	adult	228	35	0.84 (0.78, 0.88)	9	0.045 (0.016, 0.074)	1	0.005 (0, 0.014)	16	0.079 (0.041, 0.115)	3	0.015 (0, 0.033)	6	0.031 (0.006, 0.056)
Latitude > 65°N	subadult	53	5	0.89 (0.76, 0.95)	2	0.038 (0, 0.088)	2	0.046 (0, 0.107)	1	0.029 (0, 0.082)	0	-	0	-
	adult	262	38	0.86 (0.81, 0.89)	7	0.028 (0.007, 0.049)	7	0.028 (0.007, 0.049)	21	0.082 (0.048, 0.115)	1	0.004 (0, 0.013)	2	0.008 (0, 0.018)
55°N < Latitude < 65°N	subadult	66	12	0.81 (0.7, 0.89)	2	0.027 (0, 0.064)	2	0.033 (0, 0.076)	4	0.073 (0.001, 0.139)	2	0.034 (0, 0.08)	2	0.035 (0, 0.081)
	adult	332	79	0.75 (0.7, 0.79)	10	0.036 (0.014, 0.058)	42	0.148 (0.106, 0.188)	17	0.058 (0.031, 0.085)	5	0.019 (0.002, 0.035)	5	0.016 (0.002, 0.029)
Southern Norway	subadult	13	3	0.83 (0.55, 0.94)	0	-	2	0.111 (0, 0.245)	1	0.071 (0, 0.197)	0	-	0	-
	adult	110	37	0.65 (0.56, 0.74)	1	0.01 (0, 0.031)	27	0.275 (0.181, 0.358)	9	0.091 (0.032, 0.147)	0	-	0	-
Southern Norway[g]	subadult	28	8	0.7 (0.49, 0.84)	1	0.045 (0, 0.129)	3	0.118 (0, 0.236)	3	0.136 (0, 0.269)	1	0.04 (0, 0.114)	0	-
	adult	138	39	0.7 (0.61, 0.77)	1	0.009 (0, 0.026)	28	0.24 (0.159, 0.314)	10	0.08 (0.031, 0.126)	0	-	0	-
Southern Sweden	subadult	43	9	0.81 (0.66, 0.89)	2	0.042 (0, 0.098)	0	0 (0, 0)	3	0.074 (0, 0.152)	2	0.047 (0, 0.109)	2	0.049 (0, 0.113)
	adult	187	40	0.78 (0.71, 0.83)	9	0.055 (0.019, 0.089)	13	0.084 (0.039, 0.126)	8	0.048 (0.015, 0.08)	5	0.032 (0.004, 0.06)	5	0.027 (0.003, 0.05)
Baltics	subadult	9	0[h]	-	0	-	0	-	0	0 (0, 0)	0	-	0	-
	adult	17	0	-	0	-	0	-	0	0 (0, 0)	0	-	0	-
Białowieża	subadult	7	2	0.64 (0.15, 0.90)	0	-	0	-	1	0.25 (0, 0.574)	0	-	1	0.143 (0, 0.367)
	adult	35	6	0.78 (0.57, 0.90)	1	0.043 (0, 0.123)	0	-	4	0.165 (0.003, 0.3)	0	-	1	0.029 (0, 0.082)

Harz Mountains	subadult	2	0	-	0	-	0	-	0	-	0	-	0	-
	adult	11	3	0.70 (0.32, 0.89)	1	0.143 (0, 0.367)	0	-	0	-	0	-	2	0.182 (0, 0.381)
Carpathians	subadult	1	0	-	0	-	0	-	0	-	0	-	0	-
	adult	30	3	0.90 (0.71, 0.97)	0	-	0	-	3	0.103 (0, 0.208)	0	-	0	-
BBA	subadult	1	0	-	0	-	0	-	0	-	0	-	0	-
	adult	37	4	0.86 (0.66, 0.94)	0	-	0	-	3	0.106 (0, 0.215)	0	-	1	0.04 (0, 0.114)
Northwestern Swiss Alps	subadult	16	3	0.76 (0.43, 0.92)	1	0.083 (0, 0.227)	0	-	1	0.1 (0, 0.268)	1	0.077 (0, 0.211)	0	-
	adult	52	14	0.75 (0.61, 0.84)	5	0.101 (0.013, 0.181)	1	0.021 (0, 0.06)	6	0.121 (0.025, 0.208)	1	0.02 (0, 0.058)	1	0.02 (0, 0.059)
Jura Mountains	subadult	9	5	0.65 (0.35, 0.84)	2	0.149 (0, 0.32)	0	-	2	0.182 (0, 0.381)	1	0.067 (0, 0.185)	0	-
	adult	71	11	0.84 (0.72, 0.91)	2	0.03 (0, 0.071)	0	-	5	0.079 (0.01, 0.143)	2	0.033 (0, 0.077)	2	0.033 (0, 0.078)
Dinaric-southeastern Alps	subadult	8	2	0.82 (0.45, 0.95)	2	0.182 (0, 0.381)	0	-	0	-	0	-	0	-
	adult	47	2	0.95 (0.83, 0.99)	1	0.027 (0, 0.078)	0	-	1	0.02 (0, 0.057)	0	-	0	-
Balkan	subadult	0	0	-	-	-	-	-	-	-	-	-	-	-
	adult	15	0	-	-	-	-	-	-	-	-	-	-	-
Kalkalpen	subadult	0	0	-	-	-	-	-	-	-	-	-	-	-
	adult	7	0	-	-	-	-	-	-	-	-	-	-	-
Baden-Württemberg	subadult	1	0	-	-	-	-	-	-	-	-	-	-	-
	adult	3	0	-	-	-	-	-	-	-	-	-	-	-

[a]Ages classes: adult (age > 2 years) and subadult (1 < age < 2 years).
[b]Number at risk
[c]Calculated with product-limit (i.e., Kaplan-Meier) estimator. Survival probability at age T is conditional on survival until $T - 1$.
[d]Calculated with weighted product-limit estimator. Cumulative incidence at time T is the probability of an event occurring conditional on survival until T.
[e]Number of events.

[f]Parameter estimate and 95% confidence interval.
[g]Group including animals in southern Norway where lynx were locally protected from hunting quotas during the study.
[h]When $N_E = 0$, estimates were not stated.

Appendix S9 Summary statistics on slopes of the 10,000 sampled regressions for survival rates as the response and anthropogenic (H1) and hunting (H2) mortality rates as predictors.

Group	Hypothesis[a]	Test[b]	Mean[c]	SD	90% CI		Evid. ratio[d]	Prob[e]
H1: Survival vs. anthropogenic mortality	Compensatory or over-compensatory ($b < 0$)	Slope > 0	-0.678	0.283	-1.15	-0.203	0	0
	Additive or superadditive ($b > 1$)	Slope < $-S_0$[f]	0.192	0.255	-0.244	0.612	0.279	0.218
	Partially compensatory ($b > 0$)	Slope < 0	-0.678	0.283	-1.15	-0.203	Inf	1
	Partially compensatory ($b < 1$)	Slope > $- S_0$	0.192	0.255	-0.244	0.612	3.57	0.781
H2: Survival vs. hunting mortality	Compensatory or over-compensatory ($b < 0$)	Slope > 0	-0.830	0.252	-1.27	-0.451	0	0
	Additive or superadditive ($b > 1$)	Slope < $- S_0$	0.00705	0.236	-0.412	0.358	0.842	0.457
	Partially compensatory ($b > 0$)	Slope < 0	-0.830	0.252	-1.27	-0.451	Inf	1
	Partially compensatory ($b < 1$)	Slope > $- S_0$	0.00705	0.236	-0.412	0.358	1.18	0.542

[a]Refer to Appendix S4, where b is the slope coefficient of Sandercock et al. (2011).
[b]The test is used to compare the 10,000 regression slope samples under the hypothesis (e.g., $y > x$) against its alternative (e.g., $y < x$).
[c]The mean is the average result of the test. Where the test $y > x$ is reformulated as $y – x > 0$.
[d]Evidence ratio = fraction of samples consistent with the test/fraction of samples consistent with the alternative. Values close to 1 indicate a low likelihood that the hypothesis is favored over the alternative.
[e]Fraction of samples consistent with the test.
[e]S_0 is the intercept of each sample regression.

Appendix S10 Multivariate survival model comparison showing all candidate[a] models for each spatial scale and survival time scale[b].

Scales	Model[c]	DIC[d]	ΔDIC[e]	LPML[f]	ΔLPML
Landscape					
Age	comp. GRF, PC5	6438.70	0.00	-3229.46	1.96
	comp. GRF, PC1	6439.16	0.46	-3227.49	0.00
	comp. GRF, PC4	6439.70	0.99	-3228.75	1.25
	HSI GRF	6439.84	1.13	-3229.36	1.86
	comp. GRF, PC2	6441.04	2.34	-3230.97	3.47
	comp. IID, PC4	6441.71	3.01	-3232.04	4.54
	comp. GRF, PC3	6442.82	4.12	-3230.54	3.04
	base[g] GRF	6444.21	5.51	-3228.37	0.87
	comp. IID, PC1	6448.66	9.95	-3233.27	5.77
	comp. IID, PC5	6450.05	11.35	-3232.41	4.91
	comp. IID, PC2	6450.19	11.49	-3233.58	6.08
	base IID	6450.44	11.74	-3232.33	4.83
	comp. IID, PC1+2	6453.38	14.68	-3233.83	6.33
	comp. GRF, PC1+2	6454.23	15.53	-3232.34	4.84
	HSI IID	6455.39	16.69	-3233.05	5.55
	comp. IID, PC3	NA[h]	NA	NA	NA
Annual	comp. GRF, PC5	455.60	0.00	-1733.87	4.00
	comp. GRF, PC4	3455.93	0.33	-1729.87	0.00
	comp. GRF, PC1	3457.24	1.64	-1731.13	1.26
	base GRF	3457.94	2.33	-1731.14	1.27
	HSI GRF	3457.98	2.38	-1732.31	2.44
	comp. GRF, PC2	3459.90	4.30	-1732.83	2.96
	comp. IID, PC2	3461.56	5.96	-1738.42	8.54
	comp. GRF, PC3	3462.64	7.03	-1733.67	3.80
	comp. GRF, PC1+2	3463.17	7.57	-1734.71	4.84
	comp. IID, PC1	3463.55	7.95	-1736.85	6.98
	comp. IID, PC1+2	3463.91	8.30	-1738.05	8.18
	comp. IID, pc5	3464.75	9.14	-1737.42	7.55
	HSI IID	3464.77	9.16	-1738.09	8.22
	base IID	3465.11	9.51	-1735.86	5.99
	comp. IID, PC3	3465.36	9.76	-1738.66	8.79
	comp. IID, PC4	3465.72	10.1	-1736.45	6.58
Home range					
Age	comp. GRF, PC1	6434.54	0.00	-3227.12	0.24
	comp. GRF, PC1+2	6436.71	2.16	-3227.84	0.96
	base GRF	6436.98	2.44	-3226.88	0.00

	HSI GRF	6437.66	3.12	-3229.13	2.25
	comp. GRF, PC3	6438.47	3.92	-3229.03	2.15
	comp. GRF, PC2	6439.90	5.36	-3231.22	4.34
	comp. GRF, PC4	6441.09	6.55	-3228.04	1.15
	comp. GRF, PC5	6441.31	6.77	-3230.86	3.98
	base IID	6446.83	12.28	-3232.73	5.85
	comp. IID, PC1	6449.00	14.46	-3231.06	4.17
	comp. IID, PC1+2	6449.70	15.16	-3231.47	4.59
	comp. IID, PC5	6450.01	15.47	-3234.07	7.18
	comp. IID, PC3	6450.31	15.77	-3233.92	7.03
	HSI IID	6451.91	17.37	-3233.37	6.49
	comp. IID, PC4	6452.27	17.72	-3233.07	6.19
	comp. IID, PC2	NA	NA	NA	NA
Annual	HSI GRF	3452.99	0	-1729.595061	0.00
	comp. GRF, PC2	3453.24	0.25	-1730.791324	1.19
	comp. GRF, PC1	3454.92	1.93	-1730.874633	1.27
	comp. IID, PC5	3455.60	2.60	-1738.924884	9.32
	comp. GRF, PC5	3456.05	3.06	-1733.660091	4.06
	comp. GRF, PC3	3457.81	4.82	-1731.61553	2.02
	comp. GRF, PC1+2	3457.95	4.96	-1732.041617	2.44
	comp. GRF, PC4	3459.20	6.21	-1732.746766	3.15
	comp. IID, PC1	3459.44	6.45	-1735.053738	5.45
	HSI IID	3459.88	6.89	-1737.203176	7.60
	base GRF	3461.04	8.05	-1733.289326	3.69
	comp. IID, PC1+2	3463.68	10.69	-1736.237397	6.64
	comp. IID, PC3	3464.72	11.72	-1735.658798	6.06
	base IID	3465.16	12.17	-1736.305341	6.71
	comp. IID, PC2	3465.19	12.20	-1736.977437	7.38
	comp. IID, PC4	3465.24	12.25	-1736.787668	7.19

[a]Models selected with ΔDIC or ΔLPML < 2.

[b]Landscape and home range spatial scales and age and annual survival time scales.

[c]Abbreviations: comp., component, GRF, Gaussian random field frailties; PC, principal component; IID, independent and identically distributed frailties, HSI, habitat suitability index.

[d]Deviance information criterion. The smaller the DIC, the better the model quality.

[e]Models ordered by increasing DIC.

[f]Log pseudo-marginal likelihood. The larger the LPML, the better the model performance.

[g]Base covariates only: sex + season (age time scale) and sex + hunting + age class (annual time scale).

[h]Candidate model did not converge.

Appendix S11 Covariate effect estimates from the selected[a] multivariate survival models for each spatial scale and time scale[b].

Scales		Model	Term[c]	Estimate[d]	90% CI[e]	Test[f]	Evidence[g]	P[h]
Landscape								
	Age	comp. GRF, PC5	β sex: male	0.410	0.188-0.633	> female[i]	1249	0.999
			β season: hunting	1.12	0.734-1.59	> autumn[i]	Inf	1.00
			β season: spring	0.320	-0.187-0.837	> autumn[i]	6.11	0.859
			β season: summer	-0.0548	-0.470-0.377	< autumn[i]	1.48	0.598
			β season: winter	0.647	0.243-1.11	> autumn[i]	399	0.997
			β PC5	-0.00281	-0.209-0.203	< intercept	1.05	0.512
			φ GRF frailty scale	0.00423	0.00149-0.00925			
			τ2 frailty variance	0.378	0.143-0.759			
		comp. GRF, PC1	β sex: male	0.416	0.174-0.670	> female[i]	1249	0.999
			β season: hunting	1.22	0.813-1.71	> autumn[i]	Inf	1.00
			β season: spring	0.1797	-0.378-0.719	> autumn[i]	2.56	0.719
			β season: summer	-0.0802	-0.522-0.362	> autumn[i]	1.62	0.618
			β season: winter	0.639	0.256-1.08	> autumn[i]	249	0.996
			β PC1	-0.0280	-0.125-0.0842	< intercept	2.17	0.684
			φ GRF frailty scale	0.00276	0.00111-0.00542			
			τ2 frailty variance	0.494	0.174-1.06			
		comp. GRF, PC4	β sex: male	0.422	0.191-0.668	> female[i]	832	0.998
			β season: hunting	1.11	0.697-1.63	> autumn[i]	Inf	1.00
			β season: spring	0.310	-0.182-0.859	> autumn[i]	5.36	0.843
			β season: summer	-0.0322	-0.476-0.444	> autumn[i]	1.33	0.572
			β season: winter	0.637	0.218-1.12	> autumn[i]	211	0.995
			β PC4	0.0884	-0.0466-0.224	> intercept	6.34	0.863
			φ GRF frailty scale	0.00399	0.00150-0.00852			
			τ2 frailty variance	0.281	0.0744-0.705			
		HSI GRF	β sex: male	0.448	0.204-0.713	> female[i]	1110	0.99
			β season: hunting	1.21	0.788-1.70	> autumn[i]	Inf	1.00

		β season: spring	0.256	-0.254-0.840	> autumn[i]	3.60	0.782
		β season: summer	-0.00796	-0.456-0.447	> autumn[i]	1.09	0.523
		β season: winter	0.691	0.260-1.17	> autumn[i]	332	0.997
		β HSI	0.00491	-0.00380-0.0152	> intercept	3.88	0.795
		φ GRF frailty scale	0.00386	0.00167-0.00813			
		τ2 frailty variance	0.382	0.146-0.748			
	base GRF	β sex: male	0.399	0.178-0.623	> female[i]	999	0.999
		β season: hunting	1.11	0.718-1.58	> autumn[i]	Inf	1.00
		β season: spring	0.314	-0.167-0.821	> autumn[i]	6.08	0.858
		β season: summer	-0.0640	-0.496-0.370	> autumn[i]	1.42	0.588
		β season: winter	0.602	0.194-1.05	> autumn[i]	101	0.990
		φ GRF frailty scale	0.00391	0.00141-0.00779			
		τ2 frailty variance	0.240	0.0624-0.528			
Annual	comp. GRF, PC5	β age class: subadult	-0.359	-1.00-0.276	< juvenile[i]	4.45	0.816
		β age class: adult	-0.354	-1.00-0.240	< juvenile[i]	4.89	0.830
		β sex: male	0.337	0.0856-0.589	> female[i]	77.1	0.987
		β hunt. P.[j]: nonhunt.	-1.64	-4.33--0.632	< hunting[i]	Inf	1.00
		β PC5	-0.0168	-0.233-0.209	< intercept	1.28	0.562
		φ GRF frailty scale	0.00306	0.000808-0.00648			
		τ2 frailty variance	0.634	0.200-1.31			
	comp. GRF, PC4	β age class: subadult	-0.360	-1.00-0.209	< juvenile[i]	5.34	0.842
		β age class: adult	-0.295	-0.874-0.206	< juvenile[i]	4.74	0.826
		β sex: male	0.322	0.0933-0.547	> female[i]	125	0.992
		β hunt. P.: nonhunt.	-0.902	-1.53--0.435	< hunting[i]	Inf	1.00
		β PC4	0.125	-0.00313-0.261	> intercept	17.3	0.945
		φ GRF frailty scale	0.00270	0.000730-0.00623			
		τ2 frailty variance	0.381	0.108-0.805			
	comp. GRF, PC1	β age class: subadult	-0.345	-0.959-0.229	< juvenile[i]	4.76	0.826
		β age class: adult	-0.314	-0.886-0.208	< juvenile[i]	5.07	0.835
		β sex: male	0.317	0.0893-0.558	> female[i]	84.4	0.988

		β hunt. P.: nonhunt.	-0.980	-1.71--0.447	< hunting[i]	Inf	1.00
		β PC1	-0.0733	-0.142--0.00489	< intercept	25.5	0.962
		φ GRF frailty scale	0.00411	0.00133-0.00847			
		τ2 frailty variance	0.310	0.123-0.654			
	base GRF	β age class: subadult	-0.324	-0.944-0.241	< juvenile[i]	4.29	0.811
		β age class: adult	-0.301	-0.921-0.231	< juvenile[i]	4.15	0.806
		β sex: male	0.310	0.0850-0.545	> female[i]	78.3	0.987
		β hunt. P.: nonhunt.	-0.956	-1.76--0.460	< hunting[i]	Inf	1.00
		φ GRF frailty scale	0.00338	0.00106-0.00623			
		τ2 frailty variance	0.288	0.0801-0.648			
Home range							
Age	comp. GRF, PC1	β sex: male	0.400	0.158-0.659	> female[i]	269	0.996
		β season: hunting	1.10	0.710-1.56	> autumn[i]	Inf	1.00
		β season: spring	0.177	-0.283-0.624	> autumn[i]	2.82	0.738
		β season: summer	-0.110	-0.527-0.293	< autumn[i]	2.06	0.674
		β season: winter	0.553	0.141-1.01	> autumn[i]	80.9	0.987
		β PC1	-0.118	-0.224--0.0185	< intercept	49.7	0.980
		φ GRF frailty scale	0.00323	0.000508-0.00788			
		τ2 frailty variance	0.607	0.222-1.50			
	comp. GRF, PC1 + 2	β sex: male	0.403	0.164-0.653	> female[i]	262	0.996
		β season: hunting	1.17	0.776-1.63	> autumn[i]	Inf	1.00
		β season: spring	0.142	-0.311-0.616	> autumn[i]	2.26	0.694
		β season: summer	-0.0645	-0.476-0.349	> autumn[i]	1.49	0.599
		β season: winter	0.559	0.149-1.01	> autumn[i]	113	0.991
		β PC1	-0.119	-0.248--0.00726	> intercept	27.7	0.965
		β PC2	0.00440	-0.0847-0.0875	> intercept	1.25	0.556
		φ GRF frailty scale	0.00476	0.00102-0.0109			
		τ2 frailty variance	0.352	0.108-0.785			

	base GRF	β sex: male	0.410	0.189-0.648	> female[i]	554	0.998
		β season: hunting	1.13	0.727-1.63	> autumn[i]	Inf	1.00
		β season: spring	0.312	-0.199-0.855	> autumn[i]	5.70	0.850
		β season: summer	-0.0624	-0.502-0.409	> autumn[i]	1.50	0.600
		β season: winter	0.657	0.233-1.14	> autumn[i]	207	0.995
		φ GRF frailty scale	0.00221	0.000569-0.00498			
		τ2 frailty variance	0.569	0.226-1.17			
	comp. GRF, PC4	β sex: male	0.376	0.161-0.610	> female[i]	399	0.997
		β season: hunting	1.11	0.688-1.64	> autumn[i]	Inf	1.00
		β season: spring	0.397	-0.0813-0.907	> autumn[i]	10.5	0.913
		β season: summer	-0.0425	-0.491-0.417	> autumn[i]	1.34	0.574
		β season: winter	0.627	0.198-1.11	> autumn[i]	148	0.993
		β PC4	-0.0881	-0.182-0.0114	< intercept	13.0	0.928
		φ GRF frailty scale	0.00371	0.00105-0.00837			
		τ2 frailty variance	0.322	0.104-1.00			
Annual	HSI GRF	β age class: subadult	-0.360	-0.978-0.211	< juvenile[i]	5.57	0.847
		β age class: adult	-0.304	-0.828-0.213	< juvenile[i]	4.78	0.827
		β sex: male	0.340	0.110-0.587	> female[i]	105	0.990
		β hunt. P.: nonhunting	-1.01	-2.04--0.478	> hunting[i]	Inf	1.00
		β HSI	-0.00773	-0.0167-0.00108	< intercept	12.7	0.927
		φ GRF frailty scale	0.00181	0.000598-0.00392			
		τ2 frailty variance	0.846	0.282-1.75			
comp. GRF, PC2		β age class: subadult	-0.359	-0.992-0.212	< juvenile[i]	5.04	0.834
		β age class: adult	-0.299	-0.918-0.244	< juvenile[i]	4.15	0.806
		β sex: male	0.345	0.0974-0.595	> female[i]	101	0.990
		β hunt. P.: nonhunt.	-1.16	-2.08--0.570	> hunting[i]	Inf	1.00
		β PC2	0.0597	-0.0323-0.159	> intercept	6.14	0.860
		φ GRF frailty scale	0.00251	0.000372-0.00683			
		τ2 frailty variance	0.881	0.281-2.31			

comp. GRF PC1	β age class: subadult	-0.277	-0.907-0.322	< juvenile[i]	3.51	0.778
	β age class: adult	-0.282	-0.872-0.253	< juvenile[i]	3.86	0.794
	β sex: male	0.287	0.0522-0.521	> female[i]	44.0	0.977
	β hunt. P.: nonhunt.	-1.09	-2.82--0.483	> hunting[i]	Inf	1.00
	β PC1	-0.122	-0.208--0.0515	< intercept	453	0.997
	φ GRF frailty scale	0.00365	0.000667-0.00972			
	τ2 frailty variance	0.160	0.0378-0.460			

[a]Candidate models with change in deviance information criterion (ΔDIC) or change in log pseudo marginal likelihood (ΔLPML) < 2 were selected (Table 2).
[b]Covariate estimates for each scale shown for the most parsimonious selected models via ΔDIC. Models ordered by increasing DIC.
[c]Abbreviations: comp., component; GRF, Gaussian random field frailties; PC, principal component; IID, independent and identically distributed frailties, HSI, habitat suitability index.
[d]Positive and negative β coefficients indicate accelerated (i.e., shorter) and decelerated (i.e., longer) survival times, respectively, where $e^{-\beta}$ gives the multiplicative change in median survival time per unit covariate increase (acceleration factor). Within terms, the scale parameter, φ, describes the rate of decay in spatial correlations via $1 - e^{(-\varphi|\mathrm{distance}|)}$, and τ^2 is the variance in the survival time in frailty (i.e., random effect).
[e]Credible intervals of coefficients.
[f]The test indicates one-sided hypothesis tests used to estimate evidence ratios and posterior probabilities of the statements (posterior distributions [Appendices S22-S25]).
[g]The ratio between the probability the test is true and that it is false. Evidence close to 1 indicates a low likelihood that coefficients met the test hypothesis.
[h]P. the posterior probability of the estimate.
[i]Reference category.
[j]Hunting period (hunting vs nonhunting [nonhunt.]).

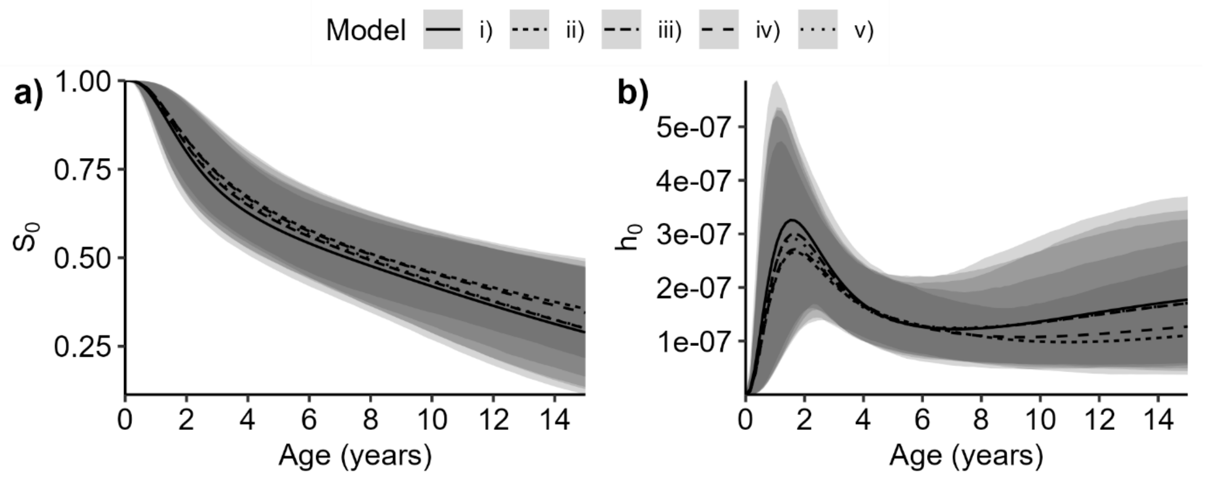

Appendix S12 Survival (a) and hazard (b) functions of the selected age time scale landscape models: i) GRF PC5, ii) GRF PC1, iii) GRF PC4, iv) GRF HSI, and v) GRF base. For covariates: season = autumn, sex = female, and median values of continuous covariates.

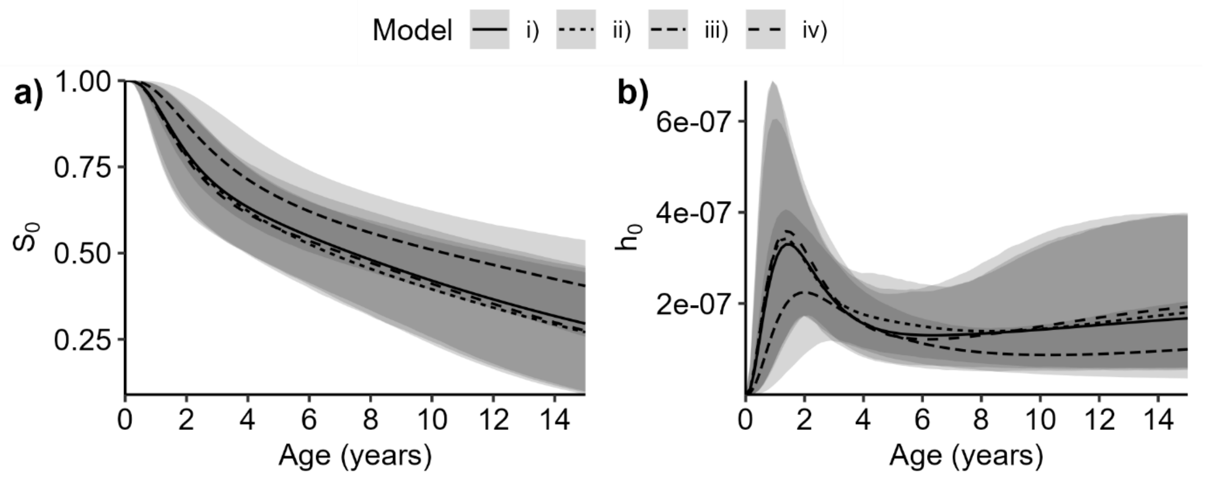

Appendix S13 Baseline survival (a) and hazard (b) functions of the selected age time scale HR models: i) GRF PC1, ii) GRF PC1 + PC2, iii) GRF base, and iv) GRF PC4. For covariates: season = autumn, sex = female, and median values of continuous covariates.

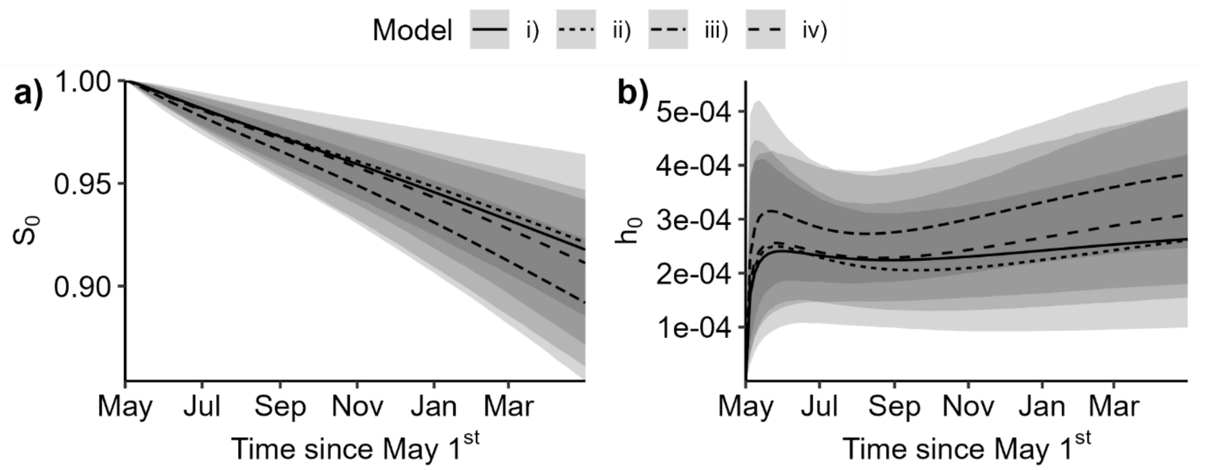

Appendix S14 Baseline survival (a) and hazard (b) functions of the selected annual time scale landscape models: i) GRF PC5, ii) GRF PC4, iii) GRF PC1, and iv) GRF base. For covariates: hunting period = nonhunting, sex = female, age class = adult, and median values of continuous covariates.

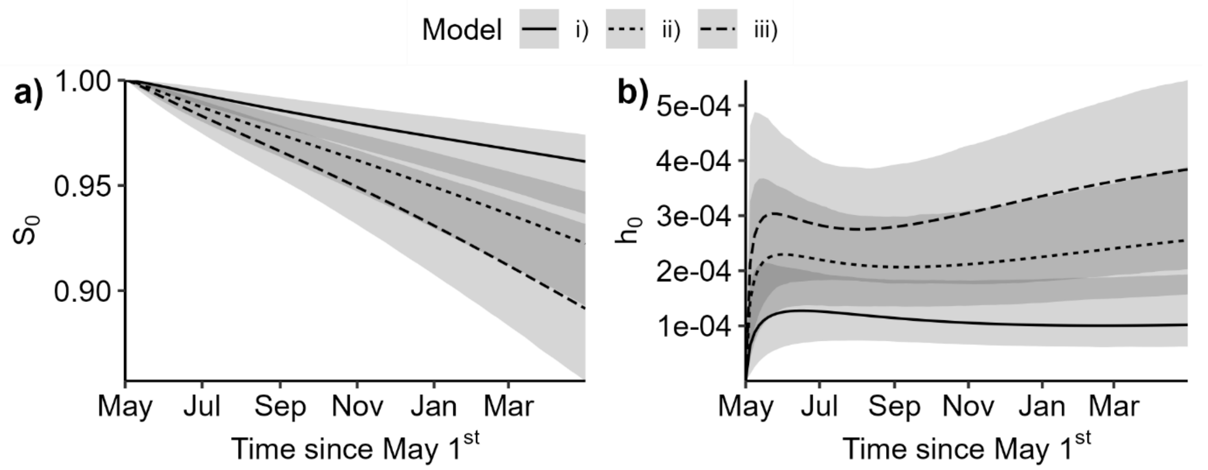

Appendix S15 Baseline survival (a) and hazard (b) functions of the selected annual time scale HR models: i) GRF HSI, ii) GRF PC2, and iii) GRF PC1. For covariates: hunting period = nonhunting, sex = female, age class = adult, and median values of continuous covariates.

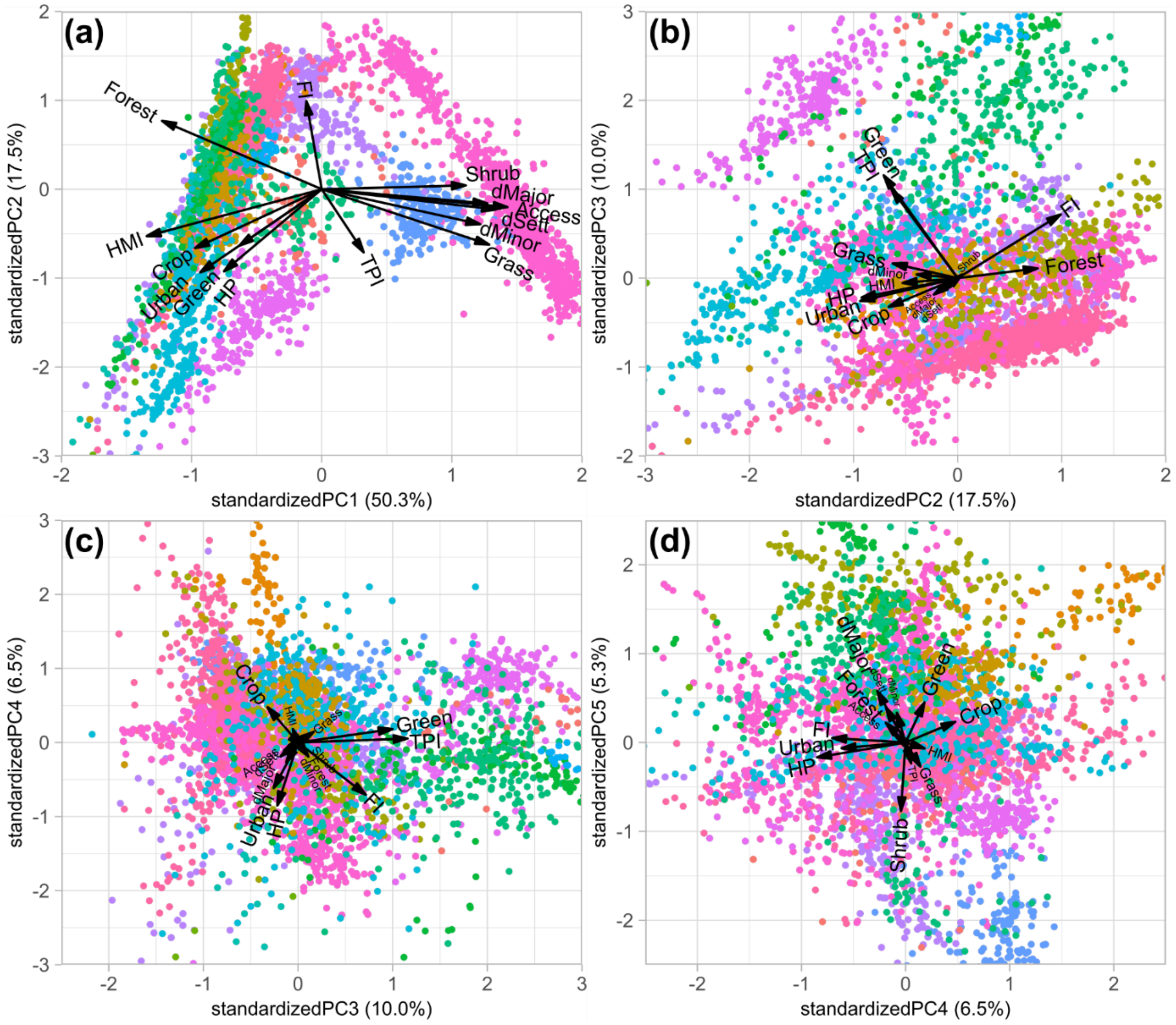

Appendix S16 Principal component analysis results for landscape scale multivariate survival model covariates. The first 5 principal components (PCs) are plotted in biplots with the more highly correlated covariates in each PC indicated with larger arrows and labels. The different colored points indicate different study areas. Plot area clipped to better visualize PC arrows. PC1 describes a gradient from human modified landscapes, including forest, with higher proportions of crop and urban land cover and higher human densities to areas with more shrub and grass land cover far from human settlements and roads (a). PC2 describes the gradient from more open grass and crop landcover in human populated areas to high integrity forested areas (a, b). PC3 describes a gradient of low to high seasonal variation in landscape greenness and low to high ruggedness (b, c). PC4 describes a gradient from higher human densities and urban cover though with higher integrity forest areas to areas with higher crop land cover (c, d). PC5 describes a gradient from high shrub land cover to high seasonality in landscape greenness.

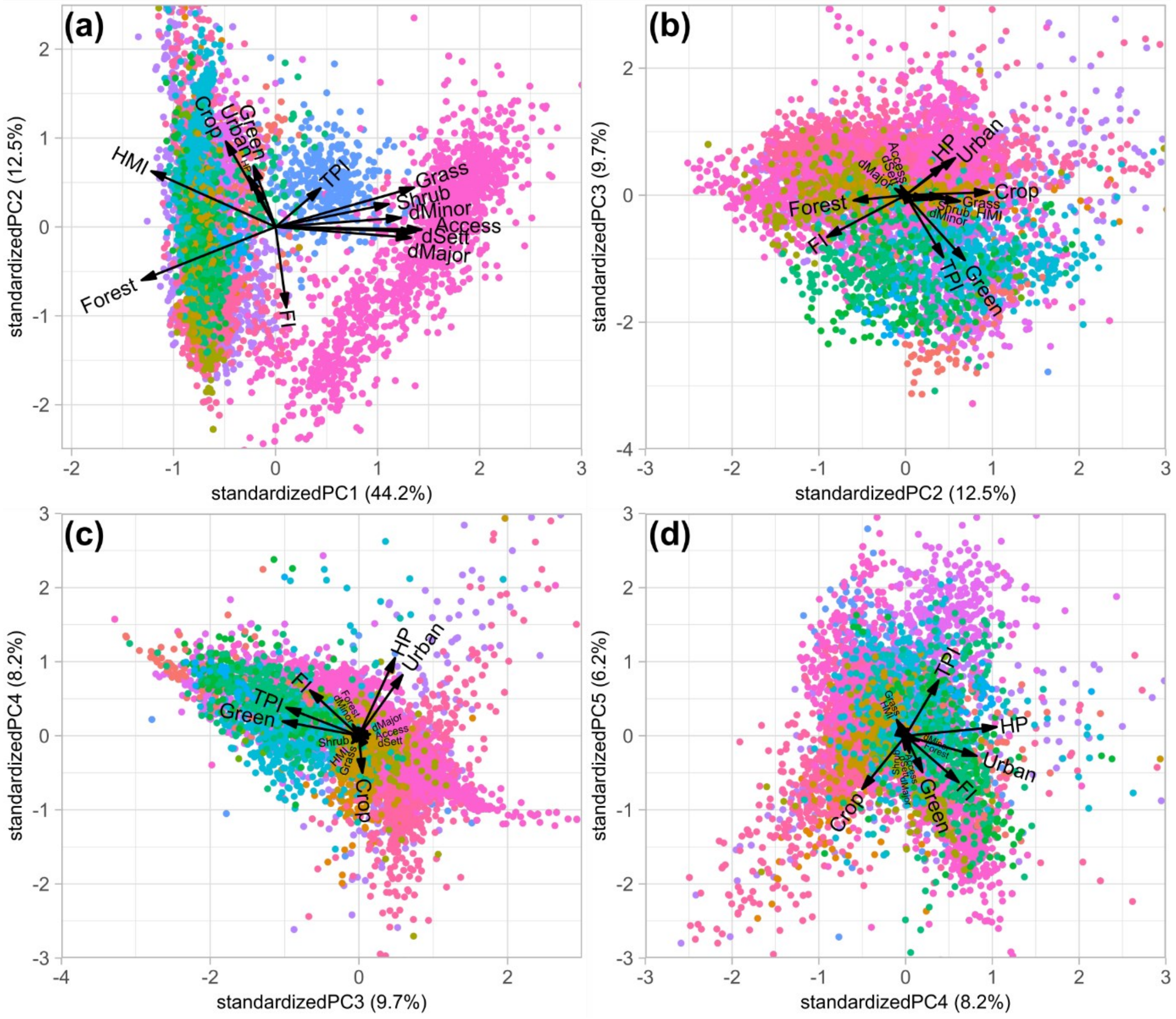

Appendix S17 Principal component analysis results for home-range scale multivariate survival model covariates. The first 5 principal components (PCs) are plotted in biplots with the more highly correlated covariates in each PC indicated with larger arrows and labels. The different colored points indicate different study areas. Plot area clipped to better visualize PC arrows. PC1 describes a gradient from more highly forested areas with high human modification to areas with more shrub and grass dominated habitats far from human settlements and roads (a). PC2 describes the gradient from high integrity forests to anthropogenic landscapes including more crops and urban land cover and higher human population densities (a, b). PC3 describes a gradient of high to low seasonal variation in landscape greenness and high to low ruggedness (mountain environment gradient (b, c). PC4 describes a gradient from high to low human population densities and urban cover (c, d). PC5 describes the gradient from low to high ruggedness and high to low crop cover (d).

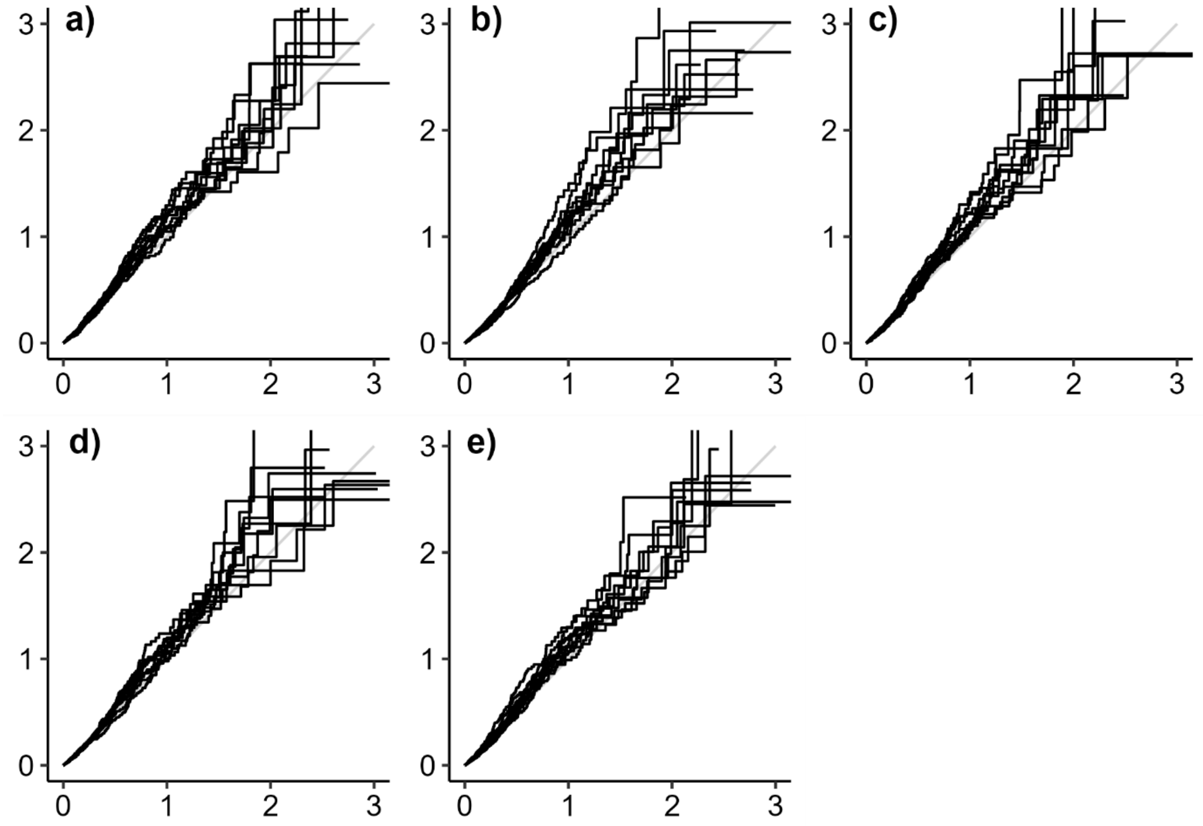

Appendix S18 Conditional Cox-Snell residual diagnostic plots of the selected age time scale landscape models: a) GRF PC5, b) GRF PC1, c) GRF PC4, d) GRF HSI, and e) GRF base. Models fit well within framework assumptions when sample curves do not depart dramatically from the grey line.

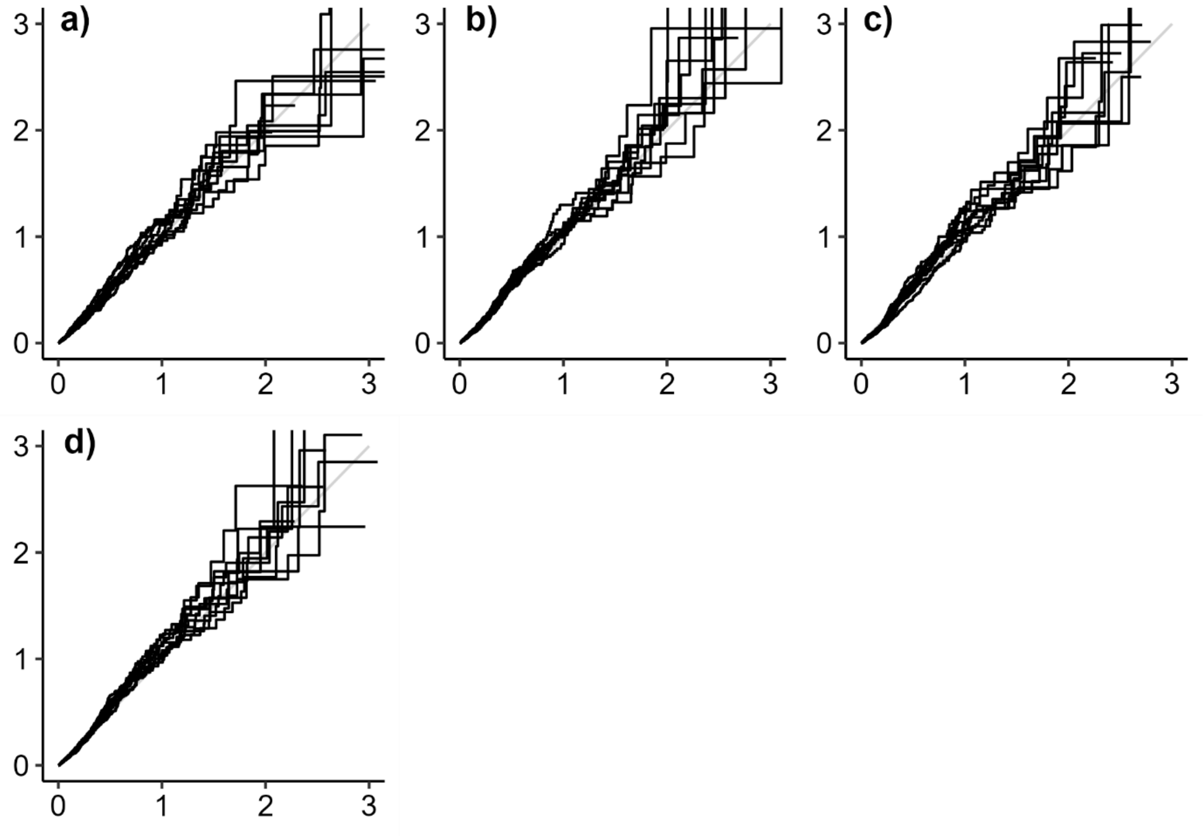

Appendix S19 Conditional Cox-Snell residual diagnostic plots of the selected annual time scale landscape models: a) GRF PC5, b) GRF PC4, c) GRF PC1, and d) GRF base. Models fit well within framework assumptions when sample curves do not depart dramatically from the grey line.

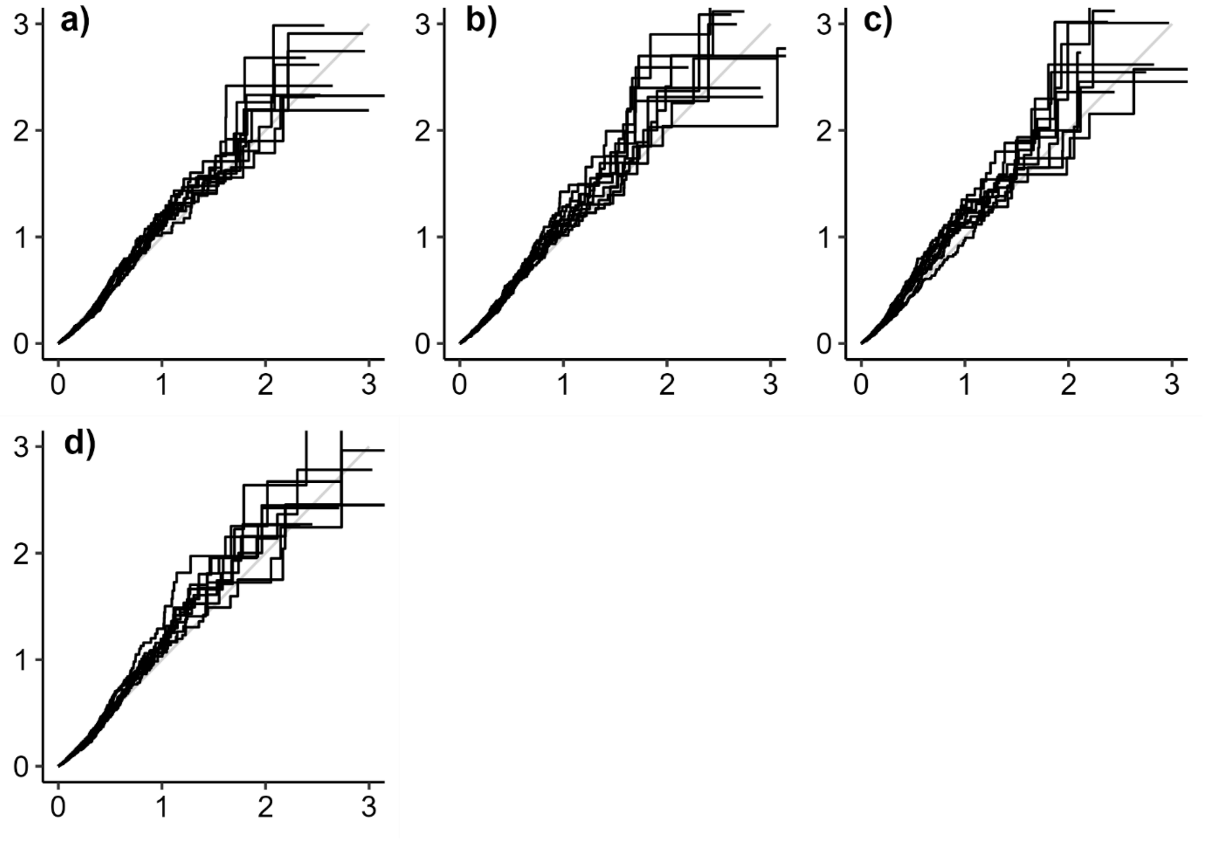

Appendix S20 Conditional Cox-Snell residual diagnostic plots of the selected age time scale HR models: a) GRF PC1, b) GRF PC1 + PC2, c) GRF base, and d) GRF PC4. Models fit well within framework assumptions when sample curves do not depart dramatically from the grey line.

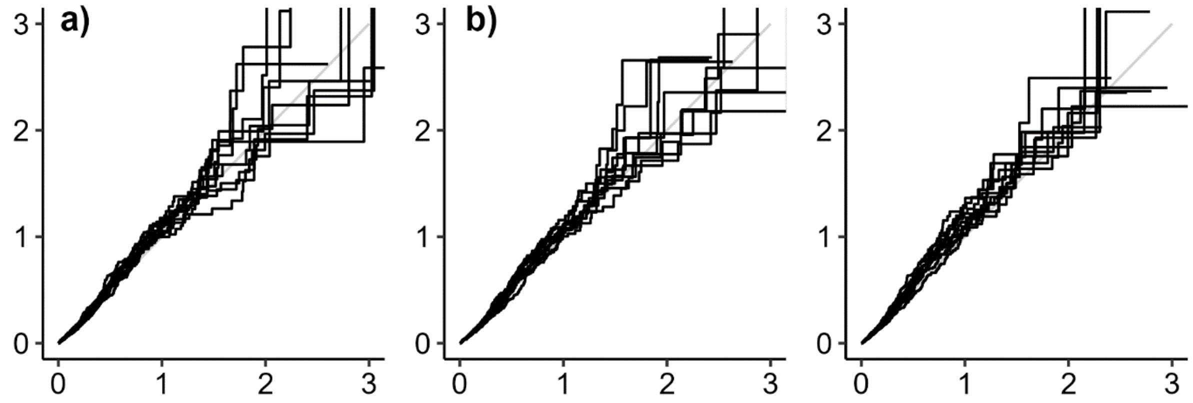

Appendix S21 Conditional Cox-Snell residual diagnostic plots of the selected annual time scale HR models: a) GRF HSI, b) GRF PC2, and c) GRF PC1. Models fit well within framework assumptions when sample curves do not depart dramatically from the grey line.

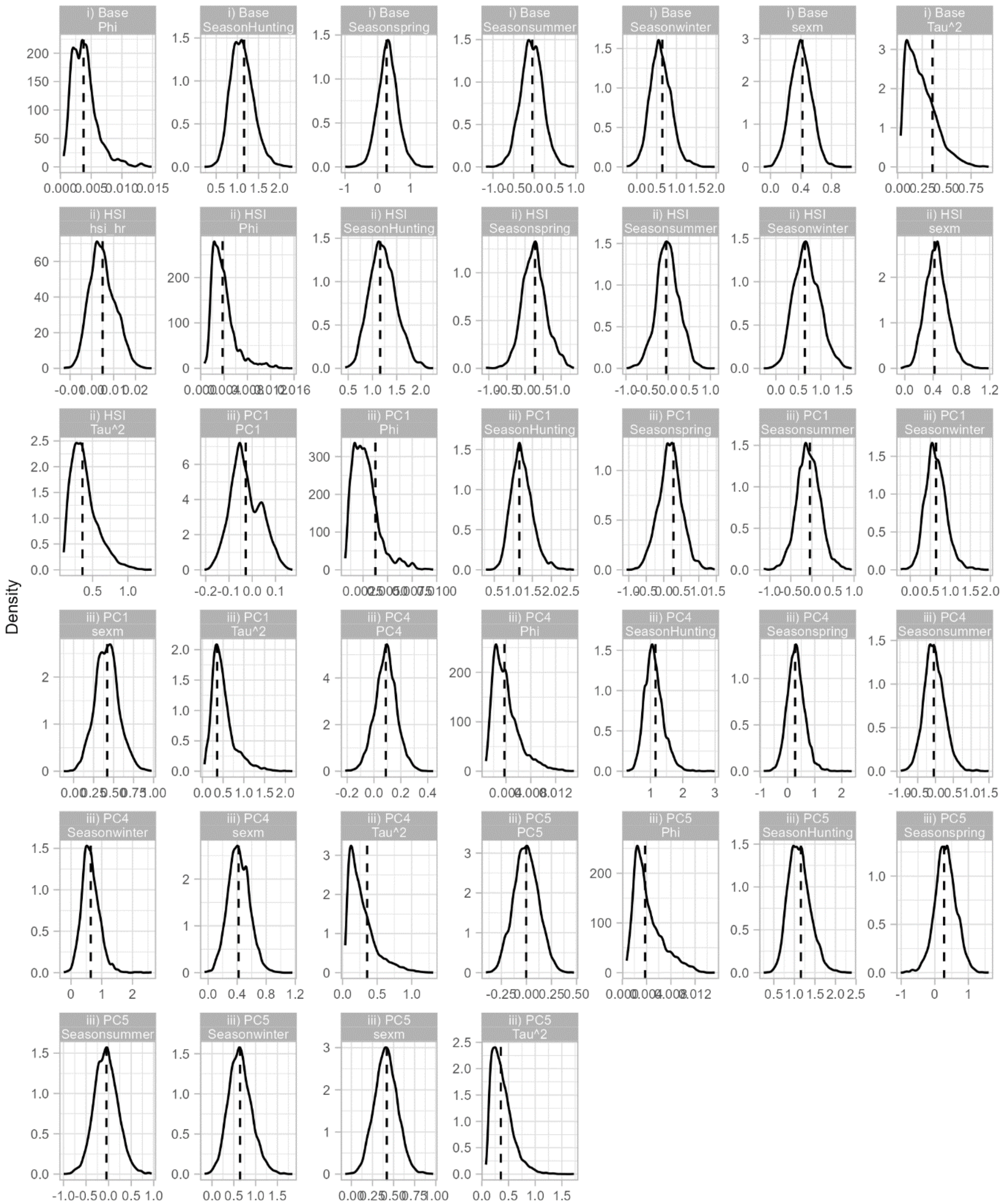

Appendix S22 Posterior distributions of coefficients from MCMC draws of the selected landscape age time scale models (all GRF frailty), with distribution means. We used noninformative default priors for model fitting.

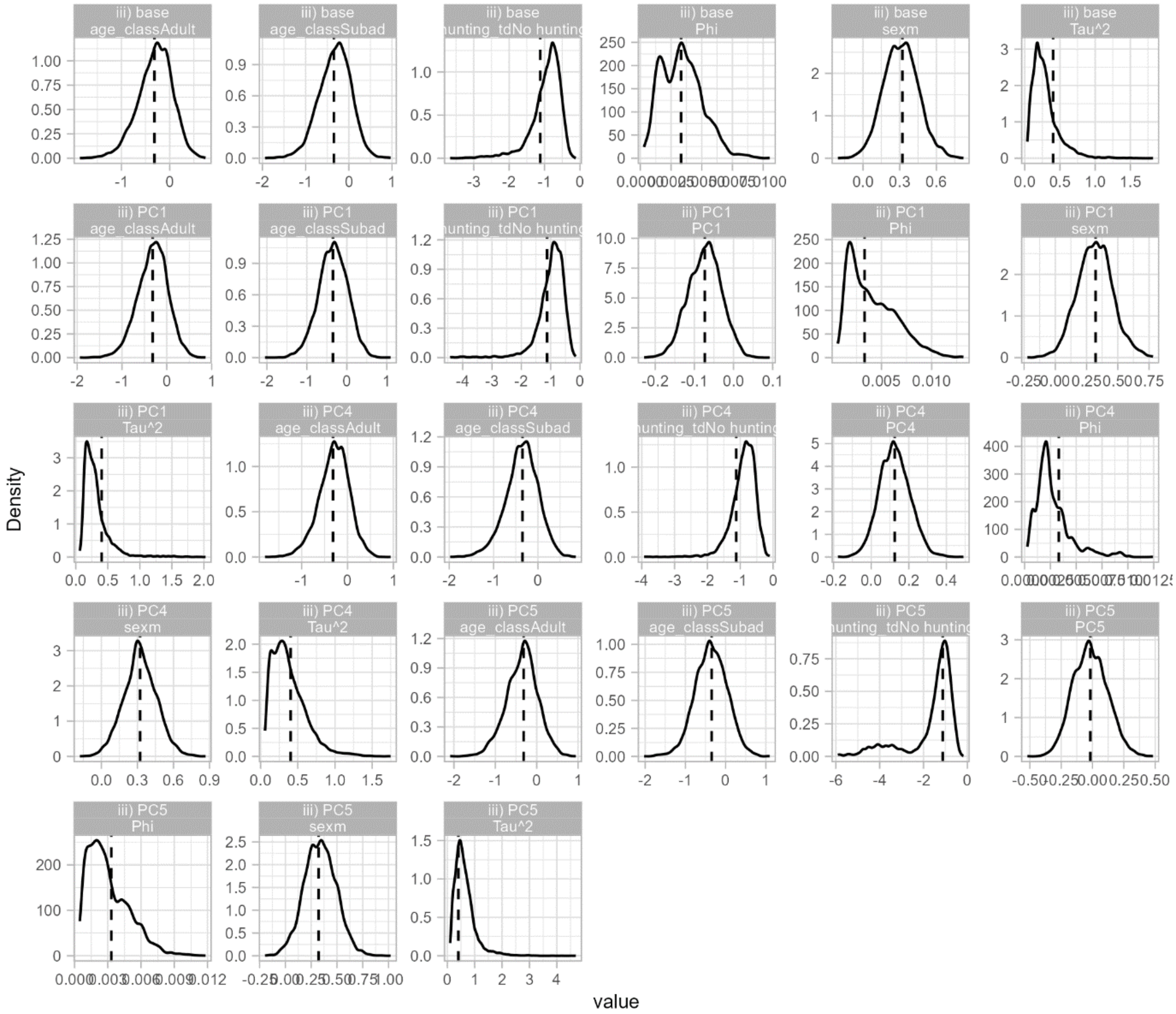

Appendix S23 Posterior distributions of coefficients from MCMC draws of the selected landscape annual models (all GRF frailty), with distribution means. We used noninformative default priors for model fitting.

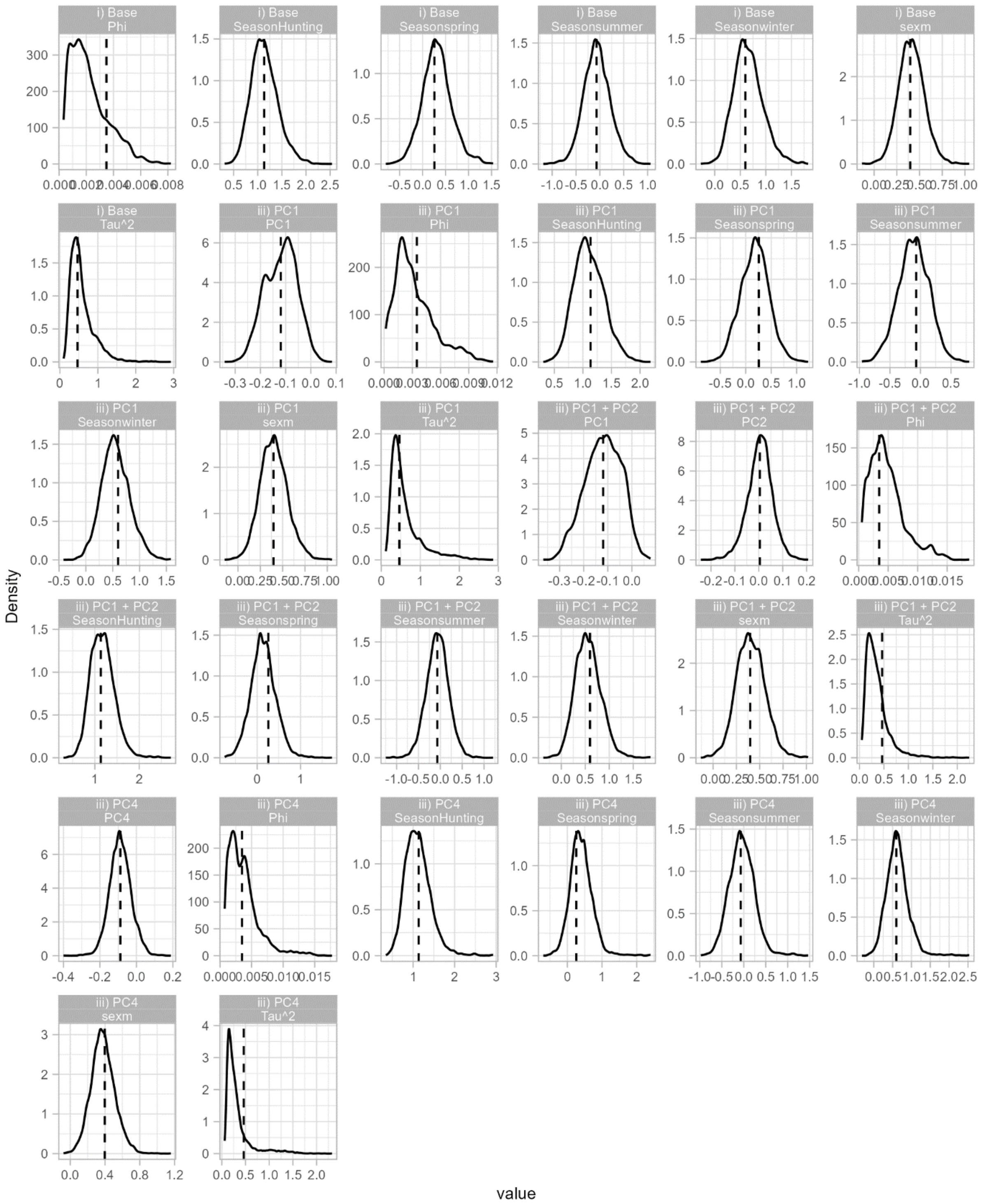

Appendix S24 Posterior distributions of coefficients from MCMC draws of the selected HR age time scale models (all GRF frailty), with distribution means. We used noninformative default priors for model fitting.

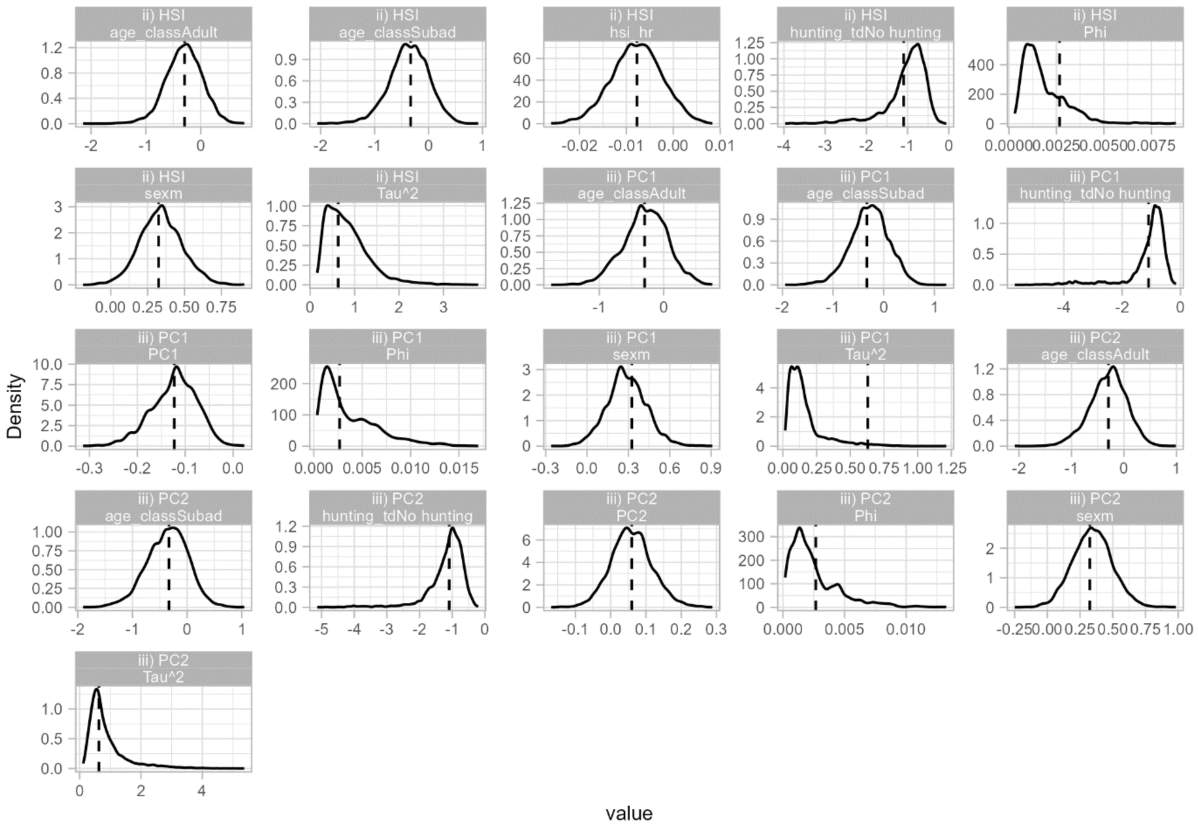

Appendix S25 Posterior distributions of coefficients from MCMC draws of the selected HR annual time scale models (all GRF frailty), with distribution means. We used noninformative default priors for model fitting.

References

Andrén, H., Linnell, J. D., Liberg, O., Andersen, R., Danell, A., Karlsson, J., … & Segerström, P. (2006). Survival rates and causes of mortality in Eurasian lynx (Lynx lynx) in multi-use landscapes. *Biological conservation*, 131(1), 23-32.

Buchhorn, M., Smets, B., Bertels, L., De Roo, B., Lesiv, M., Tsendbazar, N. E., … & Fritz, S. (2020). Copernicus Global Land Service: Land Cover 100m: Collection 3 Epoch 2015, Globe. Version V3. 0.1)[Data set].

Chapron, G., Kaczensky, P., Linnell, J. D., Von Arx, M., Huber, D., Andrén, H., … & Boitani, L. (2014). Recovery of large carnivores in Europe's modern human-dominated landscapes. *Science*, 346(6216), 1517-1519.

Farr, T. G., Rosen, P. A., Caro, E., Crippen, R., Duren, R., Hensley, S., ... & Alsdorf, D. (2007). The shuttle radar topography mission. *Reviews of geophysics*, *45*(2).

Grantham, H. S., Duncan, A., Evans, T. D., Jones, K. R., Beyer, H. L., Schuster, R., … & Watson, J. E. M. (2020). Anthropogenic modification of forests means only 40% of remaining forests have high ecosystem integrity. *Nature communications*, 11(1), 1-10.

Hofman, M. P., Hayward, M. W., Heim, M., Marchand, P., Rolandsen, C. M., Mattisson, J., ... & Balkenhol, N. (2019). Right on track? Performance of satellite telemetry in terrestrial wildlife research. *PLoS One*, *14*(5), e0216223.

Kennedy, C. M., Oakleaf, J. R., Theobald, D. M., Baruch-Mordo, S., & Kiesecker, J. (2019). Managing the middle: A shift in conservation priorities based on the global human modification gradient. *Global Change Biology*, 25(3), 811-826.

Oeser, J., Heurich, M., Senf, C., Pflugmacher, D., Belotti, E., & Kuemmerle, T. (2020). Habitat metrics based on multi-temporal Landsat imagery for mapping large mammal habitat. *Remote Sensing in Ecology and Conservation*, 6(1), 52-69.

Oeser, J., Heurich, M., Kramer-Schadt, S., Mattisson, J., Krofel, M., Krojerová-Prokešová, J., ... & Kuemmerle, T. (2023). Integrating animal tracking datasets at a continental scale for mapping Eurasian lynx habitat. *Diversity and Distributions*.

R Core Team (2022). R: A language and environment for statistical computing. R Foundation for Statistical Computing, Vienna, Austria. URL https://www.R-project.org/.

Sandercock, B. K., Nilsen, E. B., Brøseth, H., & Pedersen, H. C. (2011). Is hunting mortality additive or compensatory to natural mortality? Effects of experimental harvest on the survival and cause-specific mortality of willow ptarmigan. *Journal of Animal Ecology*, *80*(1), 244-258.

Weiss, D. J., Nelson, A., Gibson, H. S., Temperley, W., Peedell, S., Lieber, A., … & Gething, P. W. (2018). A global map of travel time to cities to assess inequalities in accessibility in 2015. *Nature*, 553(7688), 333-336.
